# *Proteus mirabilis* Vesicles Induce Mitochondrial Apoptosis by Regulating miR96-5p/Abca1 to Inhibit Osteoclastogenesis and Bone Loss

**DOI:** 10.3389/fimmu.2022.833040

**Published:** 2022-02-15

**Authors:** Tingting Wang, Lixia Mo, Jiaxin Ou, Qinghua Fang, Huimei Wu, Yuzhe Wu, Kutty Selva Nandakumar

**Affiliations:** SMU-KI International Immunopharmacology Research Center, School of Pharmaceutical Sciences, Southern Medical University, Guangzhou, China

**Keywords:** outer membrane vesicles, osteoclasts, osteoporosis, miR-96-5p, Abca1, apoptosis

## Abstract

Bone loss due to an increased osteoclast activity is common in osteoporosis and rheumatoid arthritis. For the first time, we observed an inhibition of osteoclast formation and bone resorption by outer-membrane vesicles (OMVs) from a Gram-negative, pathogenic bacterium, *Proteus mirabilis* (P.M). Gene ontogeny and KEGG enrichment analyses of miRNA and mRNA sequencing data demonstrated a significant effect of P.M OMVs on mitochondrial functions and apoptotic pathways. OMVs induced mitochondrial dysfunction through an increased level of intracellular ROS, collapse of mitochondrial membrane potential (ΔΨm), and modulation of Bax, Bcl-2, caspase-3, and cytochrome c expression. In addition, P.M OMVs strongly inhibited miR-96-5p expression, which caused an upregulation of ATP binding cassette subfamily A member 1 (Abca1) in osteoclasts leading to an increased level of mitochondria-dependent apoptosis. Moreover, treatment with P.M but not *Escherichia coli* OMVs attenuated bone loss in experimental osteoporosis and collagen-induced arthritis. Collectively, we demonstrated osteoprotective functions of OMVs from *Proteus mirabilis*, which downregulated miR-96-5p causing an increased Abca1 expression and mitochondria-dependent apoptosis.

## Introduction

Bone loss is a characteristic feature of several diseases like osteoporosis (OP), rheumatoid arthritis (RA) (1), lupus (2), Alzheimer’s disease (3), and in certain cancers (4). Bone homeostasis relies on osteoclast-dependent bone resorption and osteoblastogenesis perpetuated by osteoblasts. An increase in osteoclast numbers and function causes bone disorders (5). Fusion and terminal differentiation of mononuclear precursor cells lead to osteoclast formation, which has the unique ability to resorb bone matrix by secreting various matrix lysing enzymes (6). A balance between cell differentiation and death determines the number of osteoclasts. Therefore, bone resorption could be improved by not only reducing the formation of osteoclasts but also increasing their death rate. Apoptosis plays an important regulatory role in the osteoclast-mediated bone resorption in which a critical role for mitochondria-dependent pathways is observed (7). A shift in the regulation of osteoclast apoptosis was well documented in many osteolytic diseases including OP and RA (8). Moreover, an increased level of OC apoptosis in transgenic mice led to reduced bone resorption and osteopetrosis (9). Therefore, targeting osteoclastogenesis and osteoclast apoptosis could improve bone loss.

Bone formation and resorption also involve changes in the expression of miRNAs (21- to 23-nucleotide noncoding RNA molecules) in both osteoblasts and osteoclasts, which in turn regulate their target genes, impacting bone phenotypes (10). MicroRNAs bind to 3’-untranslated regions (3’-UTRs) on target mRNAs, which results in translational repression, target degradation, and/or gene silencing, thereby contributing to several physiological processes and disease development. Many miRNAs were reported to participate in bone regulation (9, 11, 12). For example, miR-96-5p promoted bone marrow mesenchymal stem cell proliferation and osteogenesis (13), miRNA-29b enhanced osteoclast survival by targeting Bcl-2-modifying factor (14), and miR-128 targeted the SIRT1/NF-κB signaling pathway to modulate osteoclast formation (15). Therefore, miRNAs-based therapy might be a promising approach in the treatment of osteolytic disease (9). Earlier, several miRs were shown to be involved in the regulation of pro- and anti-apoptotic genes during the activation of mitochondrial extrinsic and intrinsic apoptotic pathways (16). After apoptosis stimulation, certain miRNAs promote mitochondrial dysfunction with an increase in the cytosolic Cyto c levels and a disrupted mitochondrial membrane potential (17–19). Therefore, exploring how miRNAs affect osteoclast formation/function and their relation to osteoclast mitochondria-dependent apoptosis could be of interest.

Bacteria residing/affecting gut, oral cavity, lungs, urinary tract, and other organs can contribute to disease pathology in both RA and OP. Outer-membrane vesicles (OMVs) from such bacteria contain several components derived from it (20) that are involved in promoting its survival from harsh environmental conditions (21). These OMVs not only can interact with host cells [epithelial cells (22) and endothelial cells (23)] but also are able to modulate host immunity (24). In addition, a highly virulent *Escherichia coli* bacterium was shown to use OMVs for delivering pathogenic cargoes and directly inflicting damage to the host cells (25). Maldonado et al. (26) have reported the internalization of *Kingella kingae* OMVs by osteoblasts and synovial cells causing an increased GM-CSF and IL-6 production, which correlated with pathogen-induced bone destruction. Recently, OMVs from pathogenic *Neisseria gonorrhoeae*, *E. coli*, and *Pseudomonas aeruginosa* were shown to cause mitochondrial dysfunction by activating the intrinsic apoptotic pathway (27). Furthermore, OMV-mediated intracellular delivery of a toxin enabled an enterohemorrhagic *E. coli* to target mitochondria causing endothelial and epithelial apoptosis (28). In this context, we identified hitherto unknown osteoprotective effects of OMVs from a pathogenic bacterium, *Proteus mirabilis*, related to OP and RA that involve a specific miRNA and mitochondria-dependent apoptosis in osteoclasts.

## Methods

### Bacterial Cultures

*Proteus mirabilis* (ATCC 12453), *Escherichia coli* (ATCC 700728), *Lactobacillus casei* (ATCC 10639), and *Lactobacillus acidophilus* (CICC 6074) were from Guangdong Bioengineering Institute (Guangzhou, China) and cultured according to the ATCC protocol. Briefly, *L. casei* and *L. acidophilus* were cultured in de man, rogosa, and sharpe (MRS) broth, *P. mirabilis* was cultured in brain heart infusion broth, and *E. coli* was cultured in Luria–Bertani and nutrient broth, respectively. *L. casei* and *L. acidophilus* were grown under anaerobic conditions, while other bacteria were cultured under aerobic conditions at 37°C. All the broths were purchased from HuanKai Microbial Ltd. (Guangzhou, China).

### Isolation and Characterization of OMV

After culturing bacteria for 16–18 h at 37°C, bacteria were removed by centrifugation (8000 g, 10 min, 4°C), and the supernatant was filtered with a 0.22-μm filter (Merck Millipore, Darmstadt) to remove the remaining bacteria. OMVs were harvested by ultracentrifugation (150,000 g, 1.5 h, 4°C) and suspended in PBS for storage at -80°C until used. OMV size was measured by capturing images at 11 positions by a Nanoparticle Tracking Analyzer (Particle Metrix GmbH, Meerbusch) and analyzed using the ZetaView 8.02.31 software. OMV morphology was observed through a transmission electron microscope (Hitachi H-7500, Tokyo). A limulus amebocyte lysate chromogenic endotoxin quantitation kit (Thermofisher, Waltham) was used to check the LPS content present in Gram-negative OMVs.

### Mice

C57BL/6J (both male and female 8–10 weeks old) and DBA/1 male mice were from Guangzhou Southern Medical University Laboratory Animal Technology Development Co., Ltd. (Guangzhou, China) and Guangdong Medical Laboratory Animal Center (Guangzhou, China), respectively. All the animals were kept and bred under the same environment with a 12-h light/dark cycle. Food and water were given *ad libitum*. Animal experiments were performed by following the rules from the National Institutes of Health Guide for the Care and Use of Laboratory Animals, which were approved by the Southern Medical University Animal Ethics Board.

### Ovariectomy (OVX) and Treatment

To establish the osteoporosis model, female C57BL/6J mice (eight weeks old) were divided into 4 groups (n = 5/group), and OVX or a sham operation was done under anesthesia. Two weeks after the surgery, each mouse was injected intra-articularly with 50 μl of OMVs (5*10^8^/ml) or PBS once in a week. After 2 months, all the mice were anesthetized with isoflurane; blood samples were collected through retro-orbital plexus and sacrificed thereafter. The blood was centrifuged at 4,500 rpm at 4°C for 15 min, and the serum samples were collected and stored at -80°C until used. Femurs were collected and fixed in 4% paraformaldehyde solution.

### Collagen-Induced Arthritis (CIA)

Bovine collagen type II (Chondrex, Redmond, WA) dissolved at 2 mg/ml concentration with PBS having 0.1 M acetic acid was emulsified in 1:1 volume of Complete Freund’s adjuvant (Sigma-Aldrich, St. Louis). DBA/1 male mice (9–11 weeks old) were immunized intradermally at the base of tail (100 μl/mouse) at day 0 and boosted at day 21 but with Incomplete Freund’s adjuvant (Sigma-Aldrich). At the day of the disease onset, mice were divided into four groups: normal, CIA + PBS, CIA + P.M OMVs, and CIA + *E. coli* OMVs groups. OMVs (5*10^8^) were injected into ankle joints. Clinical arthritis was recorded in a blinded manner following a scoring protocol described earlier (29). All the mice were sacrificed 86 days after the booster. Hind paws were used for micro-CT and histology, whereas sera collected were used for CTX-1 and OCN analysis.

### ELISA

Levels of the osteoclast bone resorption maker, CTX-1, and the osteogenic activity marker, OCN, in the sera were detected by ELISA (R&D Systems, Minneapolis). CII-coated ELISA plates were used to measure anti-CII antibody levels as described earlier (30).

### Microcomputed Tomography (μCT) Analysis

A high-resolution μCT scanner (Siemens, Berlin, Germany) was used to analyze the bone quality of femurs and paws after fixing them for 48 h in 4% paraformaldehyde solution. Scanner parameters were set as follows: 80-kV voltage, 500-μA current, and 9.56-μm resolution. Trabecular bone 3D models were reconstructed with the Multimodal 3D visualization software.

### Bone Histomorphometry Analysis

Femur bones and hind paws were fixed with 4% paraformaldehyde for 48 h at room temperature (RT) and decalcified for 4 weeks in 10% EDTA solution. To examine OMV effects on the histological structure of osteoporosis mice femoral histomorphometric analysis was performed on H&E and TRAP-stained paraffin sections. Histological images were analyzed by an optical microscope (Leica, Wetzlar). Histological scores were given according to a previously published protocol (31). Briefly, the score was given by assessing the infiltration of immune cells, synovial membrane thickness, cartilage destruction, and bone erosion with a scale of 0–3 (0: normal; 1: minor; 2: moderate; 3: severe).

### Osteoclastogenesis and Transfection

Bone marrow-derived macrophages (BMMs) were extracted from C57BL/6J male mice. BMMs were isolated in cold, serum-free α-MEM (Hyclone, Logan) and cultured in a medium containing 10% fetal bovine serum (Gibco, Rockville), 2 mM glutamine, and 100 mg/ml of penicillin–streptomycin solution (Hyclone). After placing the cells in the incubator for 24 h, the supernatant was exchanged with 25 ng/ml of M-CSF (PeproTech, Rocky Hill) containing medium for 2 days, and then 50 ng/ml of RANKL (PeproTech) was added to induce osteoclast differentiation. After 5 days, TRAP staining was done, in which TRAP^+^ cells with > 3 nuclei were considered as osteoclasts.

To do suppression or overexpression of miRNA, inhibitors or mimics were used. BMMs were transfected with miR-96-5p mimic (50 nM), inhibitor (100 nM), or controls based on the riboFECTTM CT transfection protocol (RiboBio, Guangzhou).

### Proliferation Assay

After plating BMMs (5*10^4^/ml) in triplicates, the 96-well plates containing cells were incubated with various concentrations of OMVs with M-CSF for 24, 48, or 96 h. Next, after adding CCK-8 reagent (10 μl/well) and incubating for 3 h at 5% CO_2_ at 37°C, absorbance at 450 nm was measured by a Biotek reader (Winooski).

### Flow Cytometric Analysis

BMMs were cultured in M-CSF and RANKL containing medium with or without P.M OMVs for 5 days, and a BD Pharmingen™ FITC Annexin V Apoptosis Detection Kit I (BD Biosciences, San Jose) and a JC-1 mitochondrial membrane potential assay kit (Bestbio, Shanghai) were used to detect apoptosis and mitochondrial membrane potential (MMP) ΔΨm, respectively. For MMP measurement, osteoclasts were collected after treatment with P.M OMVs for 5 days, stained with JC-1 for 20 min at 37°C in dark, washed twice with 1 x FACS buffer, and analyzed by flow cytometry. ROS production was detected utilizing a ROS Assay Kit (Beyotime, Shanghai). Apoptosis, ROS, and MMP were detected and analyzed by the BD FACSCanto II (BD Biosciences) and Flow Jo 10 software (BD Biosciences), respectively. Gating strategy for FACS is given in **Figure SF6**.

### F-Actin Ring Formation

BMMs were cultured with RANKL in 24-well plates for 5 days to get OCs. After fixing with 4% paraformaldehyde for 15 min, cells were permeabilized with 0.1% TritonX-100 for 30 min and blocked with 4% BSA for 1 h. Subsequently, cells were stained with rhodamine phalloidin (Invitrogen, Carlsbad) and DAPI. Images were taken under an inverted fluorescence microscope (Carl Zeiss, Oberkochen).

### Pit Formation Assay

BMMs were cultured and induced in an osteoassay stripwell plate (Corning, St. Lowell) for 10 days containing M-CSF with or without RANKL. Cells induced by RANKL were treated with or without OMVs. After removing the adherent cells with 0.3% hypochlorous acid, wells were washed with distilled water and pits made by osteoclasts were visualized two-dimensionally in the resorption area, under an inverted fluorescence microscope, and analyzed with the Image-pro plus software. For 3D visualization, the topography of the resorbed area was reconstructed by the Image J software.

### Osteoblast Differentiation Assay

MC3T3-E1 cells (CRL-2594) from ATCC were cultured in an osteogenic medium containing α-MEM having FBS (10%), β-glycerophosphate (10 mM), L-ascorbic acid (50 μg/ml), and dexamethasone (10 ng/ml) from Sigma-Aldrich and changed once in three days. After day 7 or 14, cells were fixed with 4% paraformaldehyde for 15 min at RT and incubated with a BCIP/NBT AP substrate kit (Beyotime) for ALP staining, and the enzyme activity was quantified with an ALP test kit (Beyotime). After 21 days, 2% alizarin red stain (ARS) (Beyotime) was used to evaluate the formation of mineralized deposition.

### Scratch Assay

NIH3T3 cells (CRL-1658) from ATCC were cultured in DMEM (Hyclone). A cross-scratch was done by a 10-μl pipette tip, when cells reached the 70–80% confluent stage and then incubated with a serum-free medium with or without 5 or 0.15 μg/ml of OMVs. Images were taken at 0, 12, and 24 h under a Carl Zeiss Axio Observer inverted microscope (Oberkochen, Germany) at 40 × magnification. The area of the scratches was analyzed by the Image J software.

### RT-qPCR

Trizol^®^ reagent (Invitrogen) was used to extract the total RNA from cells and quantified using a multifunctional microplate reader. Bestar™ qPCR RT Kit (DBI, Ludwigshafen) was used for reverse transcription, and then cDNAs were used as templates for the qPCR assay (Promega, Madison) in a Roche LC480 cycler (Roche, Mannheim). β-Actin served as the mRNA internal control. Changes in relative mRNA expression were calculated by the 2^-ΔΔct^ method. All the mRNA primers are listed in **Supplemental Table ST1**.

The miRNA was quantified according to a miDETECT A Track™ qRT-PCR Starter Kit (Ribobio, Guangzhou). Briefly, after poly (A) tailing, total RNA (1 μg) was used for cDNA synthesis to do the qPCR assay. The 2^-ΔΔct^ method was used to calculate the expression of 8 microRNAs, and U6 served as an internal control. The miRNA primer sequences were purchased from RiboBio (Guangzhou, China).

### Western Blot

OCs were lysed in RIPA solution (Beyotime) to extract total proteins, which were quantified as per instructions from a BCA kit (Thermo Fisher Scientific). Protein samples (20 μg) were loaded onto 8% to 15% SDS-PAGE gel, separated, and transferred to PVDF membrane (Millipore, Shanghai). After blocking with 5% skim milk, primary antibodies against Abca1 (1:1,000, affinity, Liyang, China), Bax (1:1,000, affinity), Bcl-2 (1:1,000, affinity), beta-actin (1:3,000, Bioss, Beijing, China), Caspase-3 (1:1,000, Cell Signaling Technology), c-Fos (1:1,000, Abcam, Cambridge, UK), COX IV (1:1,000, Abbkine, Wuhan, China), CTSK (1:1,000, Abcam), Cytochrome c (1:1,000, affinity), ERK (1:1,000, Abbkine), IκB-α (1:1,000, Signalwayκ Antibody, Maryland, USA), MMP9 (1:500, ProteinTech, Wuhan, China), NFATc1 (1:1,000, Cell Signaling Technology, Danvers, USA), p-ERK (1:1,000, ProteinTech), and tubulin (1:1,000, affinity) were used to detect the target proteins. After washing for 30 min (5 min * 6 times) with TBST, corresponding secondary antibodies purchased from Abbkine (1:8000) were incubated for 2 h at RT. The images were taken through a Bio-Rad imager after developing the membrane with a HRP substrate. Band intensities were analyzed by the Image J software.

### RNAseq Analysis

To perform RNA-seq analysis, the quality of RNA was detected in agarose gel electrophoresis and NanoDrop 2000 (Thermo Scientific). The integrity of RNA was evaluated with a RNA 6000 Nano kit and an Agilent 2100 bioanalyzer (Agilent Technologies, Santa Clara). A TruSeq™ RNA sample preparation kit (San Diego, USA) was used to purify poly-A transcripts. The mRNA-seq was performed using Illumina Novaseq 6000 (San Diego, USA). Paired-end reads (2*150 bp read length/sample) were obtained on an Illumina Novaseq 6000. For quality control, raw paired end reads were trimmed utilizing SeqPrep (https://github.com/jstjohn/SeqPrep) and Sickle (https://github.com/njoshi/sickle) programs with default parameters. TopHat2 (http://tophat.cbcb.umd.edu/) was used for required mapped reads, while the Cufflinks (http://coletrapnelllab.github.io/cufflinks/) were allowed for assembly and splicing of the mapped reads. For the identification of DEGs, the transcripts per million reads (TPM) procedure was used to find the expression level of each transcript. Differential expression analysis was done with the DEseq2 program. DEGs with |log2FC| ≥ 1 and a padjust < 0.01 were taken as statistically significant. GO analysis was performed on the DAVID website. KEGG pathways were analyzed based on the KEGG pathway database.

A miRNA library was constructed by applying an Illumina TruSeq Small RNA Library preparation kit (San Diego, USA). High-throughput sequencing was carried out by Illumina Novaseq 6000. The miRNA sequence reads were matched to the miRNA sequences from mice available in the miRBase database (release 22) with the miRDeep2 software package. The miRNA expression level was calculated by TPM and analyzed by the DEseq2 program. Four computational tools (miRDB, TargetScan, RNA22, and miRWalk) were used for predicting the target mRNA of miRNAs.

### Mitochondrial Ultrastructure Analysis

BMMs (1*10^6^ cells/well) were cultured in a medium containing M-CSF and RANKL and treated using OMVs for 3 days. After harvesting, cells were centrifuged at 4°C for 5 min at 2,000 rpm. About 2.5% glutaraldehyde (Nacalai Tesque, Tokyo) was added to fix the cells at RT for 1 h and then left at 4°C for 3 h. After aspirating the fixative, cells were suspended in PBS, and TEM was used to observe the ultrastructure of mitochondria.

### Isolation of Mitochondria

Osteoclast mitochondria were obtained according to Cell Mitochondria Isolation Kit specifications (Beyotime). Briefly, M-CSF and RANKL-induced BMMs with or without P.M OMVs treatment for 5 days were washed and collected in PBS. After a mitochondria isolation reagent was added, cells were incubated for 15 min on ice and centrifuged at 4°C, 600 g for 10 min. The collected supernatant was further centrifuged at 4°C, 11,000 g for 10 min, and the cell pellets obtained were lysed with a mitochondria lysate buffer for WB analysis. The new supernatant was collected to detect the Cyto c expression in the cytoplasm.

### ATP Assay

An ATP assay kit (Beyotime) was used for the quantification of ATP levels. Briefly, after removing cell debris by centrifugation (12,000 g, 4°C, 10 min), supernatant (10 μl) was added to the ATP working solution (100 μl), and the luminescence was recorded using a microplate reader (Biotek).

### Luciferase Assay

The target site between mmu-miR-96-5p and Abca1 was predicted by TargetScan. Subsequently, Abca1 WT 3’UTR and Abca1 Mut 3’UTR were constructed (Fenghui Biotechnology, Changsha, China). The mmu-miR-96-5p mimic and the constructs, were cotransfected into HEK293T cells for 48 h, and the fluorescence was detected by following a Dual-Luciferase^®^ Reporter Assay procedure (Promega, Madison). Briefly, after lysing the cells, the supernatant was obtained by centrifugation at 4°C, 12,000 g for 5 min. A luciferase detection reagent (100 μl) was added to 100 μl of the supernatant and mixed well to detect the RLU (relative light unit). Next, an equal amount of Renilla luciferase detection working solution was added to determine the RLU. Finally, the ratio of luciferase activity at two time points was calculated, and differences were compared between the groups.

### Statistics

Quantitative data are shown as mean ± SD. Arthritis scores were given as mean ± SEM and analyzed by the GraphPad Prism 8 software (Graph Pad Software Inc., San Diego). Differences were analyzed by applying unpaired two-tailed Student’s “t” test. The p value less than 0.05 was considered to be statistically significant.

## Results

### *P. mirabilis* OMVs Inhibited RANKL-Induced Osteoclasts Compared to Other Bacterial OMVs

**Figure 1:**
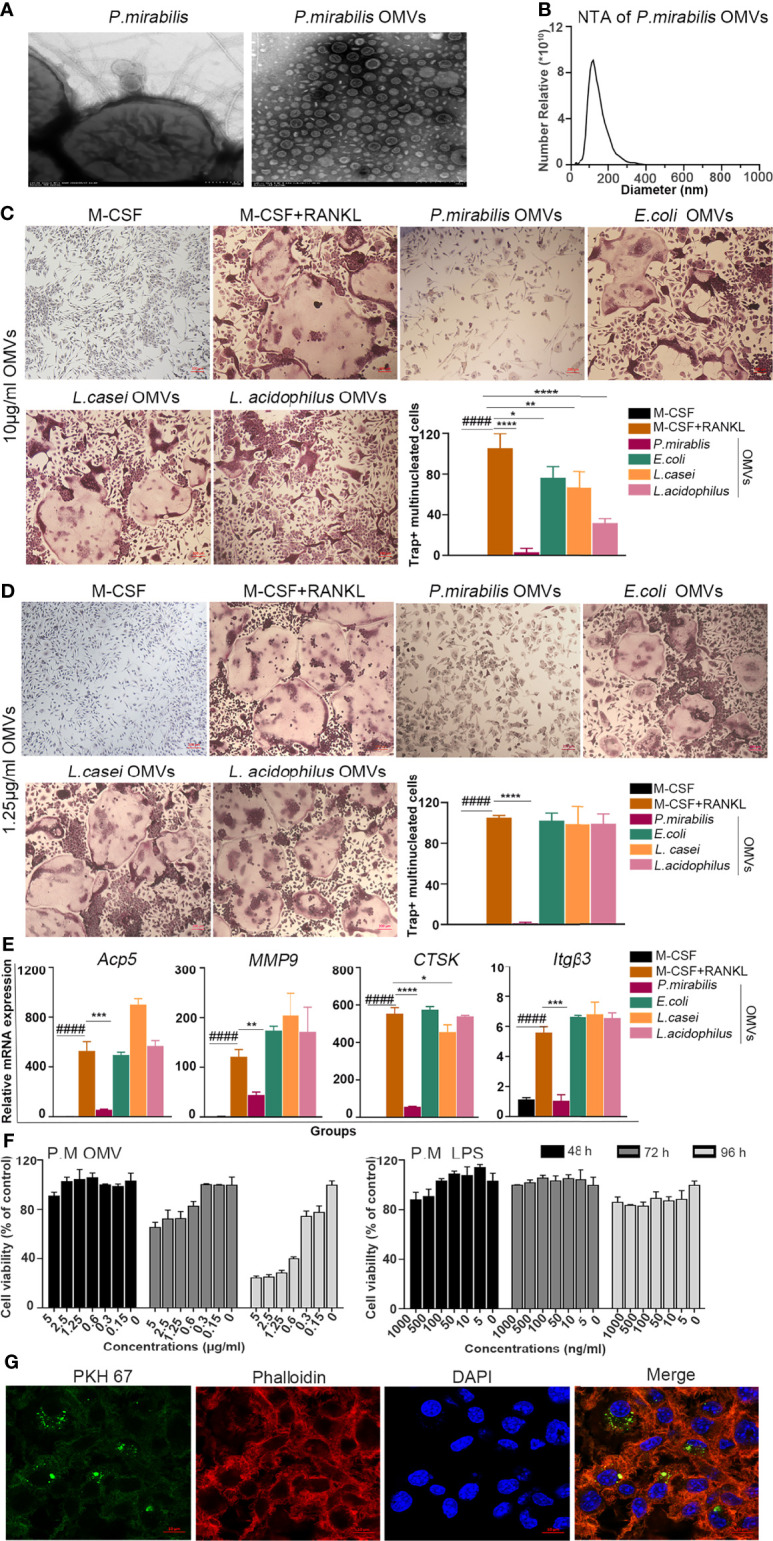
Internalized bacterial OMVs inhibited osteoclast formation and gene expression. **(A)** Morphology and **(B)** size determination of P.M OMVs by transmission electron microscopy and Nanotrack analysis. **(C)** TRAP^+^ multinucleated cells after treatment with 10 **(D)** or 1.25 μg/ml of OMVs. **(E)** Effect of *E. coli*, *P. mirabilis*, *L. casei*, or *L. acidophilus* OMVs (10 μg/ml) on the expression of osteoclast-related genes. **(F)** Effect of P.M OMVs (0.15 and 0.3 μg/ml) and standard P.M LPS (0–1,000 ng/ml) on BMM viability. **(G)** Confocal image showing internalization of P.M OMVs by osteoclasts. *E. coli*, *Escherichia coli*; *P. mirabilis*, *Proteus mirabilis (P.M)*; *L. casei*, *Lactobacillus casei*; *L. acidophilus*, *Lactobacillus acidophilus*. Scale bar 200 nm in **(A)**, 200 μm in **(D)**, and 50 μm in **(G)**. Data are from 3 independent experiments and represented as mean ± SD. ^####^p < 0.0001 compared to M-CSF; *p < 0.05; **p < 0.01; ***p < 0.001; ****p < 0.0001 compared to M-CSF + RANKL.

The contribution of bacterial outer membrane vesicles (OMVs) to host pathology and immune response is now well documented (32–34). However, how OMVs affect bone health and disease is not investigated so far. This study explored OMV modulated osteoclast differentiation (**Figures 1A–G** and **Figures S1A–C**). Based on the information from a microbe–disease interaction database (MDIDB), four bacteria related to RA and osteoporosis (*E. coli*, *P. mirabilis*, *L. casei*, and *L. acidophilus*) were chosen (**Figure S1A**). At first, the morphology and size of bacterial OMVs were determined by a transmission electron microscope and Nano track analysis (**Figures 1A, B** and **Figures S1B, C**). OMVs prepared from various bacteria were found to be homogeneous both in structure and size. Then, the effect of different OMVs on osteoclast formation was investigated. As shown in **Figures 1C, D**, different protein concentrations of OMVs (10 and 1.25 μg/ml) were used to stimulate BMMs cultured for 5 days in a medium containing M-CSF and/or RANKL with bacterial OMVs inhibited formation of osteoclasts at a high concentration. Especially, the number of Trap^+^ multinucleated osteoclasts was negligible after treatment with *P. mirabilis* OMVs (**Figure 1C**). In addition, when treated with 1.25 μg/ml, only *P. mirabilis* OMVs (P.M OMVs) have strongly reduced osteoclast formation (**Figure 1D**). Next, qPCR was done to determine the expression changes in osteoclast-related genes after treatment with different bacterial OMVs at 1.25 μg/ml. As shown in **Figure 1E**, P.M OMVs significantly reduced the expression of tartrate-resistant acid phosphatase 5 (*Acp5)*, matrix metalloproteinase 9 (*MMP9*), cathepsin K (*CTSK*), and integrin subunit β3 (*itgβ3*) genes. In summary, these data demonstrated a strong suppression of osteoclast differentiation by P.M OMVs.

*P. mirabilis* is a Gram-negative bacterium, and its OMVs contain lipopolysaccharide (LPS). In the present study, the LPS content of P.M OMVs was detected using a limulus amebocyte lysate assay, and a microgram of protein was found to have 1.76 ± 0.06 ng of LPS. Thereafter, different concentrations of P.M OMVs (0.15–10 μg/ml) or standard P.M LPS (0–1,000 ng/ml) were used to stimulate BMMs for 48, 72, and 96 h, and the cell viability was assessed using a cell counting kit 8 (CCK8). P.M OMVs affected BMM viability in a dose-dependent manner, whereas P.M LPS (0–1,000 ng/ml) had only a milder effect (**Figure 1F**).

Bielaszewska et al. (23) reported an uptake of EHEC O157 OMVs by cells involved in the pathogenesis of enterohemorrhagic *E. coli*-mediated disease. In accordance with this observation, we found the internalization of PKH67-labeled P.M OMVs by osteoclasts after incubation for 6 h (**Figure 1G**).

### P. M OMVs Decreased OC Differentiation, Fusion, and Resorption Activity

**Figure 2:**
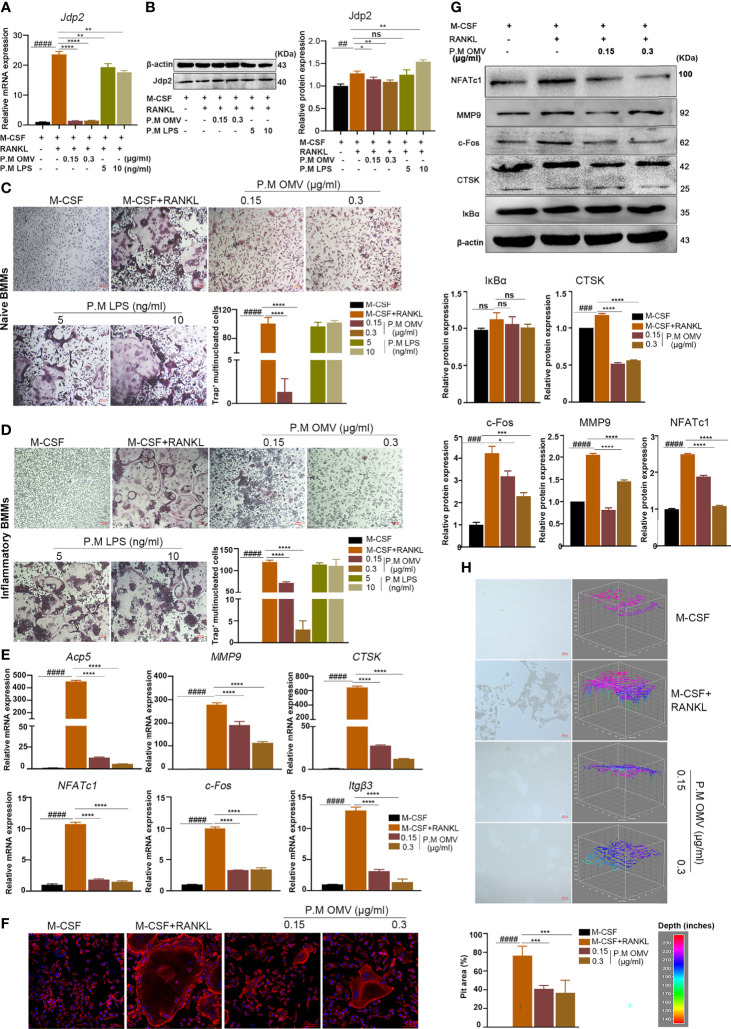
Effect of P.M OMVs on osteoclast genes, proteins, and function. **(A)** Effect of P.M OMVs (0.15 and 0.3 μg/ml) and LPS (5 and 10 ng/ml) on Jdp2 gene and **(B)** protein expression. Differentiation of **(C)** naive and **(D)** inflammatory OCs after treatment with P.M OMVs (0.15 and 0.3 μg/ml) or P.M LPS (5 and 10 ng/ml). P.M OMVs inhibited the expression of **(E)** OC-related (*Acp5, MMP9, CTSK, NFATc1, c-Fos*, and *itgβ3*) genes, **(F)** actin ring formation, **(G)** expression of proteins (MMP9, CTSK, NFATc1, c-Fos, and IkBα) and **(H)** bone resorption (n = 6). n indicates number of mice. P.M, *Proteus mirabilis*. Scale bar 200 μm in **(C, D, H)** and 50 μm in **(F)**. Data are from 3 independent experiments and represented as mean ± SD. ^##^p < 0.01; ^###^p < 0.001; ^####^p < 0.0001 compared to M-CSF; *p < 0.05; **p < 0.01; ***p < 0.001; ****p < 0.0001 compared to M-CSF + RANKL. ns, not significant.

Jun dimerization protein 2 (Jdp2), a repressor protein of AP-1, has a higher level of expression in neutrophil-mediated antibacterial immunity as well as in RANKL- but not LPS-induced osteoclastogenesis (35, 36). In this study, Jdp2 expression was found to be significantly upregulated after RANKL treatment. Importantly, P.M OMVs (0.15 and 0.3 μg/ml) but not standard P.M LPS (5 and 10 ng/ml) have significantly inhibited Jdp2 expression (**Figures 2A, B**). Next, we hypothesized that such an inhibition of Jdp2 expression by P.M OMVs but not standard P.M LPS might also suppress osteoclast formation of BMMs derived from both naive and inflamed mice, which was proved to be correct (**Figures 2C, D**).

F-actin ring maintains the morphology and function of osteoclasts. When F-actin structure is damaged or unable to form, it affects osteoclast formation and bone resorption. Hence, the effect of P.M OMVs on the F-actin cytoskeletal structure was evaluated using confocal microscopy. As shown in **Figure 2F**, mature osteoclasts had intact F-actin belts with multiple nuclei after RANKL treatment; however, P.M OMVs decreased the size of OCs while increasing the number of nuclei by disrupting the F-actin ring structure. Furthermore, P.M OMVs have also significantly reduced the resorption area of OCs compared to the RANKL-treated group (**Figure 2H**). These data demonstrated the ability of P.M OMVs in inhibiting the differentiation and function of osteoclasts. In addition, P.M OMVs inhibited the expression of OC-related genes (*Acp5, c-Fos, CTSK, itgβ3, MMP9*, and *NFATc1*) and proteins (c-Fos, CTSK, MMP9, and NFATc1 except IkBα) (**Figures 2E, G**).

Several cells including osteoclasts, osteoblasts (OB), and fibroblasts are involved in the maintenance of bone homeostasis. Therefore, we tested the effect of P.M OMVs on osteoblasts and fibroblasts as well. P.M OMVs had negligible effect on osteoblast (MC3T3-E1) proliferation (**Figure S2A**). However, alkaline phosphatase (ALP) staining, ALP activity level, and alizarin red staining (ARS) indicated moderate effect of P.M OMVs on OB differentiation by downregulating the expression of *BSP*, *OCN*, and *Runx2* genes, although OPN gene expression was significantly increased (**Figures S2B–E**). On the other hand, CCK8 and scratch assays demonstrated a negligible effect of P.M OMVs on the viability (**Figure S2F**) and migration (**Figure S2G**) of fibroblasts.

### The miRNA and RNA Expression Analysis After P.M OMV Treatment

**Figure 3:**
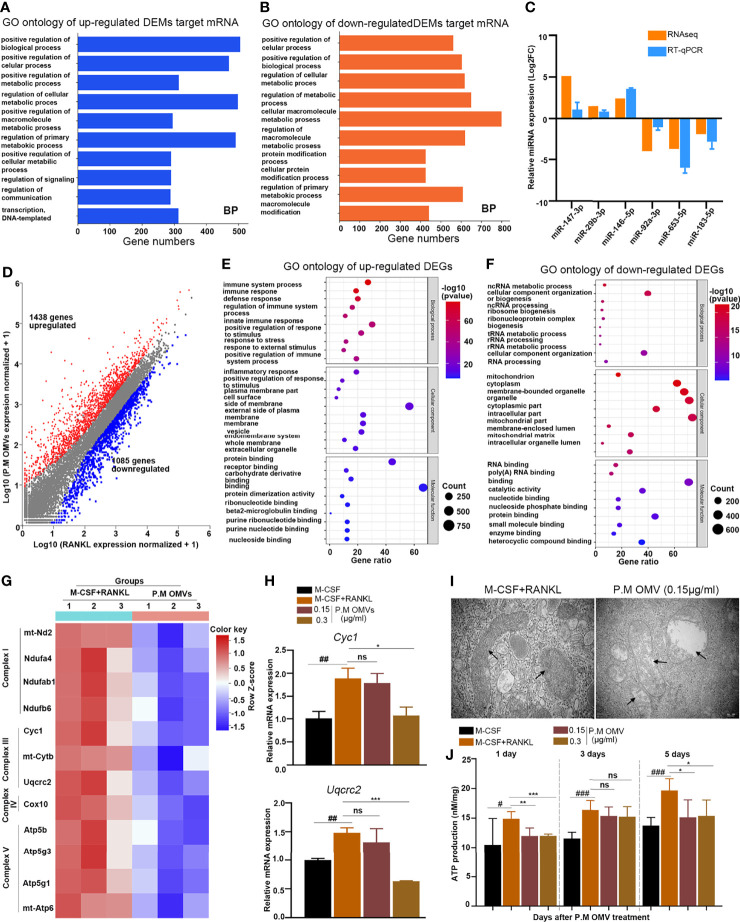
The miRNA and RNA sequence analysis after P.M OMV treatment. **(A, B)** GO category analysis of up- and downregulated miRNA targeted mRNAs in the P.M OMV group for biological process. The top ten enriched significant GO categories (p < 0.01) are shown. **(C)** Validation of miRNA-seq data by RT-qPCR (n = 6). **(D)** Comparison of expressed genes after treatment with P.M OMVs and RANKL. **(E, F)** The top ten GO enrichment analysis of up- and downregulated mRNAs in the P.M OMV group for biological process, cell component, and molecular function, respectively. **(G)** Heatmap of mitochondria-related genes after RANKL or RANKL + OMV treatment. **(H)** Verification of mitochondria-related genes by RT-qPCR. **(I)** Representative TEM images of mitochondrial morphology in osteoclasts. **(J)** ATP production after RANKL with or without 0.15 μg/ml P.M OMV treatment for 5 days. P.M, *Proteus mirabilis*. The samples for miRNA and mRNA sequencing were took from triplicates. ^#^p < 0.05; ^##^p < 0.01; ^###^p < 0.001. *p < 0.05; **p < 001; ***p < 0.001 compared to M-CSF + RANKL. ns, not significant.

To explore how P.M OMVs affect osteoclasts at a molecular level, small RNA (miRNA) and mRNA sequencing were performed. Identification of differentially expressed miRNAs (DEMs) by miRNA-sequencing (miRNA-seq) showed 44 upregulated and 48 downregulated (|log_2_FC| ≥ 1.0 and P value < 0.01) miRNAs in P.M OMV-treated cells (**Figure S3A**). Gene Ontology (GO) enrichment analysis of miRNA target genes in the P.M OMV group showed an association with mitochondrial functions (**Figures 3A, B**). Next, we randomly selected 6 miRNAs and detected their expression by RT-qPCR, which confirmed miRNA-seq data (**Figure 3C**).

In the RNA-seq analysis, 1,438 genes were upregulated and 1,085 genes were downregulated (|log_2_FC| ≥ 1.0 and P value < 0.01) after P.M OMV treatment (**Figure 3D**). Gene Ontology (GO) enrichment analysis of mRNAs in the P.M OMV group showed an association of upregulated DEGs with cellular immunity (**Figure 3E**) and downregulated DEGs to mitochondrial functions (**Figure 3F**). Next, we have drawn a heatmap with the 12 downregulated genes associated with mitochondria (**Figure 3G**). Subsequently, the expression of mitochondria-related genes (*Cyc1* and *Uqcrc2*) was detected by RT-qPCR and found to be decreased after P.M OMV treatment (**Figure 3H**), which confirmed the RNA-seq data.

TEM analysis of OC mitochondria after P.M OMV treatment was found to be swollen and lacked cristae, whereas in the RANKL group, mitochondria displayed canonical tubular patterns (**Figure 3I**). In addition, we found that P.M OMVs have significantly decreased ATP production at days 1 and 5 in RANKL-induced osteoclasts (**Figure 3J**). These data demonstrated the P.M OMV ability to cause mitochondrial dysfunction and downmodulate ATP synthesis in osteoclasts.

### P.M OMVs Promoted Mitochondria-Dependent Apoptosis in Osteoclasts

**Figure 4:**
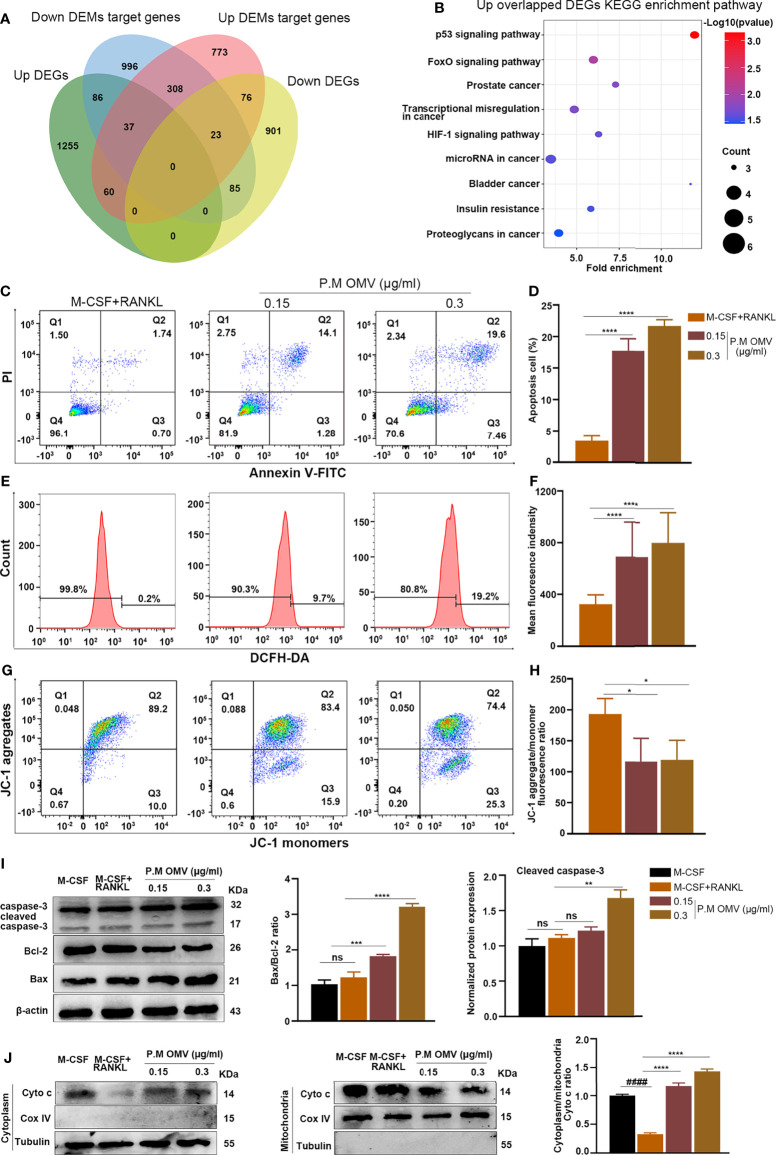
Effect of P.M OMVs on mitochondria-dependent osteoclast apoptosis. **(A)** Venn diagram showing the intersection of negatively correlated miRNA–mRNA pairs within miRNA and mRNA sequences. **(B)** KEGG enrichment analysis of upregulated genes is shown in the bubble chart. The top 15 genes significantly enriched in the KEGG pathway (p < 0.05) are presented. Changes in osteoclast **(C, D)** apoptosis, **(E, F)** intracellular ROS, and **(G, H)** mitochondrial membrane potential level after being cultured with or without 0.15 or 0.3 μg/ml of P.M OMVs for 5 days. **(I)** Western blot analysis of caspase-3, Bcl-2, and Bax. **(J)** Cyto c expression in the cytoplasm and mitochondria with or without P.M OMV treatment. Data were obtained from three independent experiments and represented as mean ± SD. ^####^p < 0.0001 compared to M-CSF. *p < 0.05; **p < 001; ***p < 0.001; ****p < 0.0001 compared to M-CSF + RANKL. ns, not significant.

To further explore the interaction of miRNA–mRNA in P.M OMV-treated osteoclasts, we predicted the target genes of DEMs from miRNA-seq and performed an intersecting analysis with DEGs from RNA-seq to identify genes that were inversely coexpressed with miRNAs. In total, 1,277 and 1,535 target genes from up- and downregulated miRNAs were screened using the miRWalk database. Consequently, 123 up- and 99 downregulated genes were identified to have at least one negatively regulated miRNA–mRNA pair in P.M OMV-treated osteoclast-related DEMs (**Figure 4A**). Subsequently, these genes were selected for KEGG enrichment analysis, and the most enriched up- and downregulated DEGs were found to be involved with apoptotic pathways and cancer, respectively (**Figure 4B** and **Figure S3B**).

Interestingly, an upregulated intracellular reactive oxygen species (ROS) production from mitochondria was reported to be beneficial for osteoclast differentiation, but an excessive ROS can enhance osteoclast apoptosis (7, 37). Therefore, in order to investigate the potential mechanisms involved in P.M OMV-induced decrease in OC formation and function, we examined the effect of P.M OMVs on cell apoptosis and ROS production. Osteoclasts were treated with 0.15 and 0.3 μg/ml of P.M OMVs for 3 days before staining with Annexin V-FITC or DCFH-DA to detect apoptosis and ROS level, respectively. As shown in **Figures 4C–F**, P.M OMVs induced a higher level of apoptosis and ROS in a dose-dependent manner. Intracellular ROS accumulation contributes to changes in the mitochondrial membrane potential (MMP, ΔΨm), and a decrease in the MMP level implies the destruction of mitochondria (37, 38). To determine whether P.M OMVs affect MMP levels, we measured ΔΨm using JC-1 fluorescence probe using flow cytometry and found a significant dissipation of ΔΨm after P.M OMV treatment compared to the RANKL group (**Figures 4G, H**).

Importantly, the expression of caspase-3 and Cyto c has an important function in mitochondria-dependent apoptosis, and the mitochondrial dysfunction often leads to Cyto c release from the mitochondria (39). Therefore, we investigated the effects of P.M OMVs on the Bax/Bcl-2 ratio and the cleaved caspase-3 level, which were found to be significantly increased compared to the RANKL group (**Figure 4I**). After treatment with P.M OMVs, Cyto c was found to be significantly released from the mitochondria (**Figure 4J**). Thus, P.M OMVs were found to promote mitochondria-dependent osteoclast apoptosis.

### Inhibition of miR96-5p Promoted Mitochondria-Dependent Apoptosis

Next to construct the miRNAs–DEGs network, we used 123 upregulated intersecting genes (**Figure 4A**) and found 177 negatively regulated miRNA–mRNA pairs (**Figure S4**). Among the identified miRNAs, miR-182-5p and miR-96-5p had higher fold changes in miRNAseq data and high connection with target genes. The expression of miR-96-5p had the highest level (44 times) of bold difference (**Figure S5A**). Therefore, miR-96-5p was chosen for subsequent investigations.

**Figure 5:**
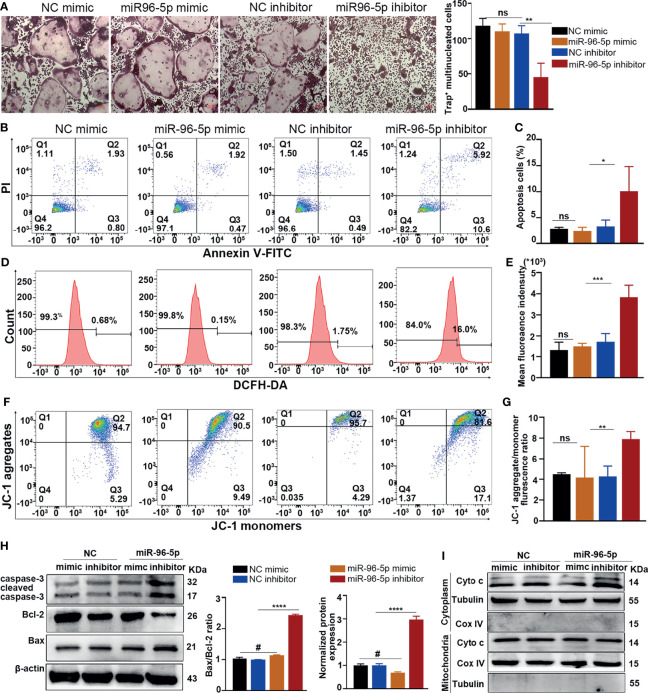
Effect of mmu-miR-96-5p on OC differentiation and apoptosis. **(A)** Effect of mmu-miR-96-5p mimic or inhibitor on OC formation. **(B, C)** Osteoclast apoptosis, **(D, E)** ROS production, and **(F, G)** mitochondrial membrane potential level after miR-96-5p mimic or inhibitor treatment. **(H)** Western blot analysis of caspase-3, Bcl-2, and Bax. **(I)** Cyto c expression in the cytoplasm and mitochondria with or without P.M OMV treatment. P.M, *Proteus mirabilis*. Scale bar = 200 μm. Data are from 3 independent experiments and given as mean ± SD. ^#^p < 0.05 compared to NC mimic. ****p < 0.0001 compared to M-CSF+RANKL. *p < 0.05; **p < 0.01; ***p < 0.001; ****p < 0.0001 compared to the NC inhibitor. ns, not significant.

Firstly, we transfected miR-96-5p mimic to BMMs and confirmed the transfection efficiency by RT-qPCR (**Figure S5B**). Transfection with the miR-96-5p inhibitor has significantly decreased osteoclast formation and the expression of OC-related genes. On the other hand, transfection with miR-96-5p mimic had an opposite effect (**Figure 5A** and **Figures S5C, D**). Inhibition of miR-96-5p promoted apoptosis in cancer cells, but its role in osteoclast apoptosis has not been reported. In this study, inhibition of miR-96-5p attenuated cell viability (**Figure S5E**) and promoted apoptosis and ROS production in osteoclasts (**Figures 5B–E**). Also, the MMP level was obviously decreased after transfection with the miR-96-5p inhibitor (**Figures 5F, G**). In addition, transfection of the miR-96-5p inhibitor increased the Bax/Bcl-2 ratio and caspase-3 expression in osteoclasts (**Figure 5H**) and also enhanced Cyto c release from the mitochondria (**Figure 5I**). These results demonstrate an increase in mitochondrial-dependent apoptosis in OCs through the inhibition of miR-96-5p.

### Inhibition of miR-96-5p by P.M OMVs Upregulated Abca1 Expression Involved in Mitochondria-Dependent Apoptosis

**Figure 6:**
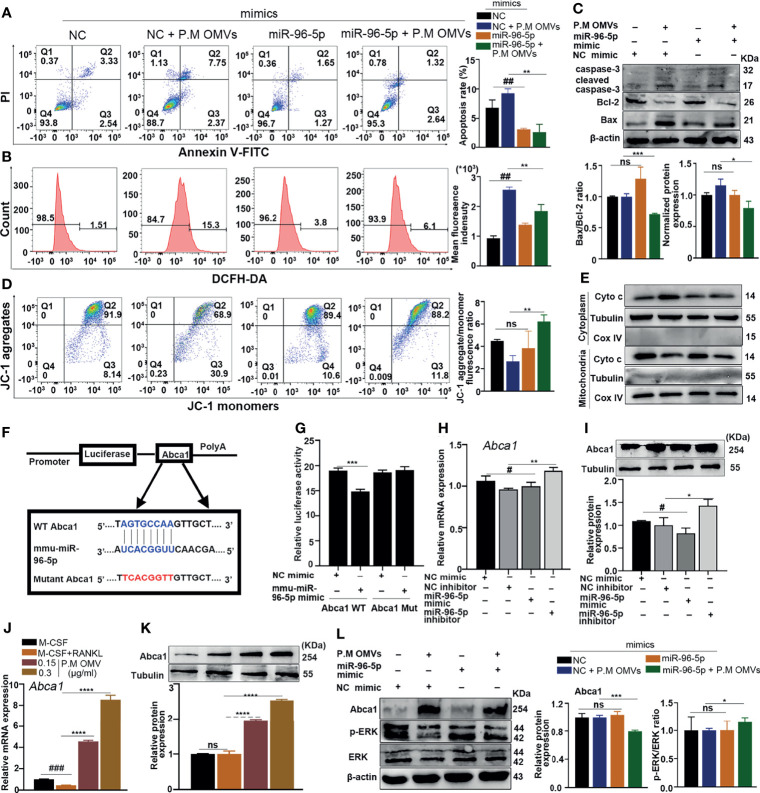
Effect of P.M OMVs on miR96/Abca1 mediated mitochondria-dependent apoptosis. **(A)** Osteoclast apoptosis, **(B)** ROS production, **(C)** Western blot analysis of caspase-3, Bcl-2, and Bax, **(D)** mitochondrial membrane potential level, and **(E)** Cyto c expression in the cytoplasm and mitochondria after mimic transfection with or without P.M OMV treatment. **(F)** Schematic diagram of miR-96-5p target site in the 3’UTR of Abca1 mRNA and its mutated sequence. **(G)** Relative firefly luciferase activity normalized to Renilla luciferase activity was shown. **(H, I)** Expression of Abca1 after NC and miR-96-5p mimic or inhibitor transfection by RT-qPCR and WB. **(J, K)** Expression of Abca1 after 0.15 μg/ml of P.M OMV treatment by RT-qPCR and WB. **(L)** Effect of miR-96-5p mimic on P.M OMVs mediated Abca1 and p-ERK expression. P.M, *Proteus mirabilis*. Data are from 3 independent experiments and given as mean ± SD. ^#^p < 0.05; ^##^p < 0.01; ^###^p < 0.001 compared to NC mimic or M-CSF. *p < 0.05; **p < 0.01; ***p < 0.001; ****p < 0.0001 compared to M-CSF + RANKL or NC mimic + P.M OMVs. ns, not significant.

Next, we found that in P.M. OMV-induced apoptosis, ROS production was counteracted after transfection with miR-96-5p mimic, which demonstrated that P.M OMV-induced apoptosis was indeed through miR-96-5p (**Figures 6A, B**). Also, the protein level of cleaved caspase-3 was significantly decreased (**Figure 6C**). In addition, miR-96-5p also attenuated P.M OMV-induced Cyto c release and collapse of MMP (**Figures 6D, E**). These results demonstrated that the downregulation of miR-96-5p caused by P.M OMVs has induced osteoclast apoptosis.

As a next step, miRDB, TargetScan, RNA22, and miRWalk were used to predict the potential target genes of miR-96-5p by overlapping all the available target genes with the 123 upregulated DEGs. As shown in **Figure S7**, four target genes were identified: ATP binding cassette subfamily a member 1 (Abca1), musculoaponeurotic fibrosarcoma (Maf), Neuroligin 2 (Nlgn2), and zinc fingers and homeoboxes 2 (Zhx2). Among them, Abca1, an essential gene for the efflux of cholesterol from cells, had the highest fold change (6 times), so Abca1 was chosen for subsequent studies.

Firstly, to investigate whether miR-96-5p can directly target Abca1, we transfected miR-96-5p with either a wild-type (WT) or mutant (Mut) Abca1-psiCheck2 vector into HEK293T cells (**Figure 6F**). Luciferase activity of Abca1 WT transfected cells was decreased significantly upon treatment with miR-96-5p mimic, while a negligible change in the luciferase activity was observed after Abca1-Mut transfection, demonstrating that Abca1 is indeed a target for miR-96-5p (**Figure 6G**). Then we tested Abca1 expression by RT-qPCR and WB after miR-96-5p mimic or inhibitor transfection. Abca1 expression was obviously increased by the inhibitor, while miR-96-5p mimic had an opposite effect (**Figures 6H, I**). These results demonstrated a negative regulation of Abca1 expression by miR-96-5p.

To observe whether P.M OMVs can affect the Abca1 level, we detected Abca1 expression after P.M OMV treatment. As shown in **Figures 6J, K**, Abca1 was significantly upregulated by P.M OMVs. Studies have shown that an upregulated Abca1 expression can inhibit the ERK pathway, which contributes to mitochondrial dysfunction-induced apoptosis (40–42). Here, we found that miR-96-5p mimic has decreased P.M OMV-induced upregulation of Abca1 and increased ERK activation (**Figure 6L**). Thus, inhibition of miR-96-5p by P.M OMVs could enhance the expression of Abca1 leading to an enhanced osteoclast apoptosis and destruction.

### P.M OMVs Improved Bone Loss in Ovariectomy (OVX) Induced Osteoporosis

**Figure 7:**
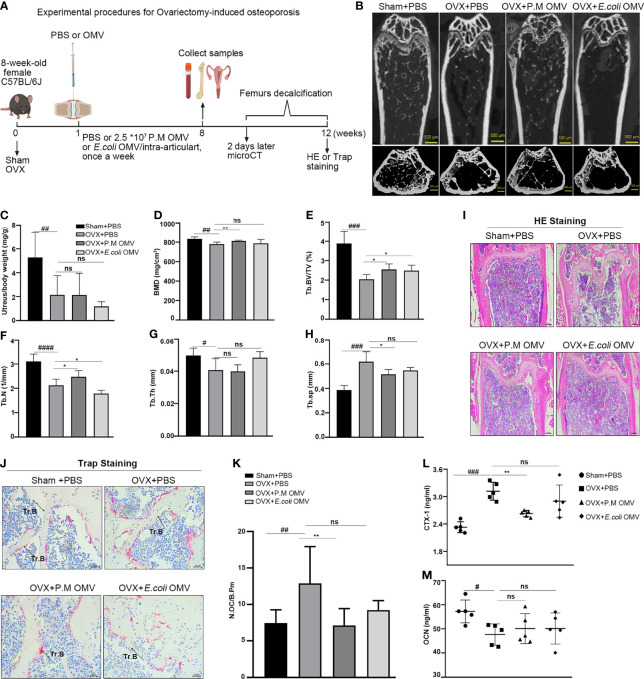
P.M OMVs protected against OVX-induced bone loss. **(A)** Experimental protocol. **(B)** MicroCT images of sham and OVX (with and without P.M or *E*. *coli* OMV treatment) operated mouse femurs. **(C)** Uterine to body weight ratio **(D–H)** BMD, Tb.BV/TV, Tb.N, Tb.Th, Tb.Sp measurements **(I)** HE (n = 5) and **(J)** TRAP (n = 5) stained femur sections and **(K)** N.Oc/B.Pm ratio. Serum **(L)** CTX-1 and **(M)** OCN levels (n = 5/group). n indicates the number of mice. BMD, bone mineral density; Tb.BV/TV, trabecular bone volume per tissue volume; Tb.N, trabecular number; Tb.Th, trabecular thickness; Tb.Sp, trabecular separation. Sham, OVX, OVX + P.M OMV, and OVX + *E*. *coli* OMV (n = 5/group) groups were used for μCT scanning experiments. n indicates the number of mice. P.M, *Proteus mirabilis*. Scale bar 500 μm in **(B)**, 200 μm in **(I)**, and 25 μm in **(J)**. Data are from 2 independent experiments and given as mean ± SD. ^#^p < 0.05; ^##^p < 0.01; ^###^p < 0.001; ^####^p < 0.0001 compared to Sham. *p < 0.05; **p < 0.01 compared to OVX. ns, not significant.

In osteoporosis and RA, disruption in the homeostatic regulation of osteogenic bone formation and osteoclast bone resorption leads to bone loss. Therefore, next we explored the function of P.M OMVs on bone protection *in vivo*. An OVX-induced osteoporosis model was established and used as shown in **Figure 7A**. OVX mice were treated with P.M or *E. coli* OMVs, or PBS through intra-articular injection, once a week. After 2 months, femurs were collected and bone phenotypes were analyzed. The trabecular bone became sparse in the OVX group, which was improved after treatment with P.M but not *E. coli* OMVs (**Figure 7B**). Successful removal of ovaries was confirmed by determining the uterus/body weight ratio (**Figure 7C**). Bone mineral density (BMD) and other trabecular bone parameters (Tb.BV/TV, Tb.N, Tb.Th) were significantly decreased in the OVX mice treated with PBS (**Figures 7D–H**). These conditions were improved after treatment with P.M OMVs. In addition, P.M OMVs narrowed OVX-induced increase in trabecular spacing in the bone (Th.sp). On the other hand, *E. coli* OMVs had moderate effects on OVX-induced osteoporosis than P.M OMVs. H&E staining confirmed the reduction in the number and thickness of trabecular bone (**Figure 7I**). TRAP staining revealed more N.Oc/B.Pm in the OVX + PBS group, which was decreased after P.M OMV treatment (**Figures 7J, K**). The marker for bone resorption, CTX-1, was significantly reduced after treatment with P.M but not *E. coli* OMVs (**Figure 7L**). However, there was no significant difference in the osteocalcin (OCN) levels because of the treatments (**Figure 7M**). Similarly, we observed that P.M but not *E. coli* OMVs have moderately improved bone loss in collagen-induced arthritis (CIA) as shown in **Figure S8A**. Although P.M. OMVs affected joint inflammation at an initial phase (**Figures S8B–D**), they improved bone erosion during the late phase of the disease (**Figures S8E, F**). A decrease in CII antibody and CTX-1 levels and an increase in the OCN level were observed (**Figures S8G–I**). Interestingly, microCT evaluations showed that P.M OMVs can improve certain bone parameters affected by arthritis (**Figures S8J–O**). Collectively, these data demonstrated the osteoprotective effects of P.M OMVs in both OVX- and CIA-induced bone loss.

## Discussion

We have analyzed the effects of OMVs from a pathogenic bacterium *Proteus mirabilis* on osteoclasts at three (*in vitro*, *in vivo*, and molecular) levels and demonstrated how a pathogenic bacterium could protect against bone loss. *In vitro*, we found that P.M OMVs inhibited osteoclast differentiation and function, moderately impaired osteoblast maturation, but had a negligible effect on fibroblasts. *In vivo*, bone loss induced in both OVX-induced osteoporosis and CII-induced arthritis was markedly attenuated after treatment with P.M but not *E. coli* OMVs. At the molecular level, i) P.M OMVs promoted mitochondrial dysfunction and induced osteoclast apoptosis; ii) based on mRNAseq and miRNAseq analysis, miR96-5p was found to have an important role in the osteoprotective functions of P.M. OMVs; and iii) inhibition of miR96-5p has increased Abca1 expression and caused osteoclast apoptosis. Thus, P.M OMVs promoted mitochondria-dependent osteoclast apoptosis and inhibited osteoclast differentiation by modulating the miR95-5p/Abca1 axis, which improved bone loss.

Outer-membrane vesicles have a critical function in disseminating virulence factors for Gram-negative pathogens (43, 44). Based on their immunomodulatory properties, these vesicles can be used for developing vaccines and in many bioengineering applications (45, 46). For the first time, we demonstrated the osteoprotective functions of vesicles from a pathogenic bacterium, *P. mirabilis*, associated with both OP and RA, in which an activation of osteoclasts and bone resorption is far exceeding than bone formation. Bacteria can affect host immunity and disease processes by producing short-chain fatty acids, toxins, and OMVs, which can pass through various physiological barriers. *Lactobacillus acidophilus* and *Lactobacillus casei* were earlier shown to improve the bone quality in OVX-induced osteoporosis (47), while *Porphyromonas gingivalis* promoted alveolar bone resorption (48). Interestingly, the introduction of segmented filamentous bacteria (SFB) into germ-free mice induced an increase in the intestinal Th17 cells (49), which potentiated arthritis development (50). SFB-induced increase in IL-17A and lipocalin-2 levels promoted osteoclasts while suppressing osteoblasts (51). Recently, exposure of macrophages to OMVs from a pathogenic bacteria was shown to activate intrinsic mitochondrial apoptotic pathways and NLR Family Pyrin Domain Containing 3 (NLRP3) inflammasomes (27). However, there is no report that studied the effect of OMVs on osteoclasts. Therefore, we evaluated OMVs from different RA- and OP-related bacteria on osteoclastogenesis and identified the osteoprotective functions of *P. mirabilis* OMVs.

It is well known that neutrophils are the first line of defense against bacterial infection, and bacteria tend to downregulate the transcription factor from the AP-1 family, Jdp2, which inhibited neutrophil maturation (36). Interestingly, modulation of Jdp2 expression also led to osteosclerosis by affecting osteoclast differentiation (35, 36). P.M OMVs inhibited the expression of Jdp2 and osteoclast-related genes and proteins, as well as impaired F-actin ring formation, which are needed to maintain the osteoclast morphology and function. So, inhibition of Jdp2 could be one of the ways by which P.M OMVs inhibited osteoclast differentiation and function. Conversely, P.M OMVs moderately affected osteoblast maturation while having a negligible effect on fibroblast proliferation and migration. These moderate effects on osteoblasts suggest that it is possible to use P.M OMVs for modulating bone homeostasis in the clinics only after the identification of active component(s) that are directly involved in osteoclast inhibition. However, if specific genes, miRNAs, and pathways affected by P.M OMVs can be identified, that might be useful for drug development to treat osteolytic diseases.

Due to the significant inhibition of P.M OMVs in osteoclast differentiation and function, RNAseq analysis was done to investigate the molecular mechanisms involved in it. GO analysis of mRNAseq and miRNAseq data showed a significant effect of P.M OMVs on osteoclast mitochondrial functions. During osteoclast differentiation, energy (ATP) requirement mainly comes from mitochondria, and its dysfunction will certainly limit ATP production (52). TEM images clearly demonstrated P.M OMV-induced mitochondrial destruction, which led to reduced ATP production. Earlier, oxidative damage to mitochondria was shown to significantly increase ROS production, which in turn induced apoptosis (53–55). Of interest, dihydroartemisinin had significantly increased the ROS level in LPS-stimulated osteoclasts, which affected the mitochondria-dependent apoptotic pathway leading to reduced osteoclast formation and bone loss (56). Based on our findings, we overlapped DEGs from mRNAseq analysis and DEMs targeted genes to do KEGG enrichment analysis, and found an upregulation of apoptotic pathways by P.M OMVs, which was confirmed further with the following observations: an increased ROS level, apoptosis rate, Bax/Bcl-2 ratio, caspase-3 expression, and Cyto c release from mitochondria. Since mitochondria are the prime source of ROS, they are more prone to an increased ROS exposure leading to enhanced apoptosis with damaging consequences (57) Earlier, Bcl-2 expression was shown to inhibit OC apoptosis by repressing Cyto c released from the mitochondria. Importantly, Bcl-2-deficient mice had a reduced number of OCs, which showed an increase in the expression of activated caspase-3 suggesting that the absence of Bcl-2 increases OC apoptosis (58) Apart from this intrinsic pathway, mitochondria-dependent apoptosis also has an extrinsic pathway involving death receptors and cytokines. Although OCs express death receptors like Fas/FasL and TRAIL/DR4, their role in OC apoptosis is uncertain similar to TGF-β (59).

**Figure 8:**
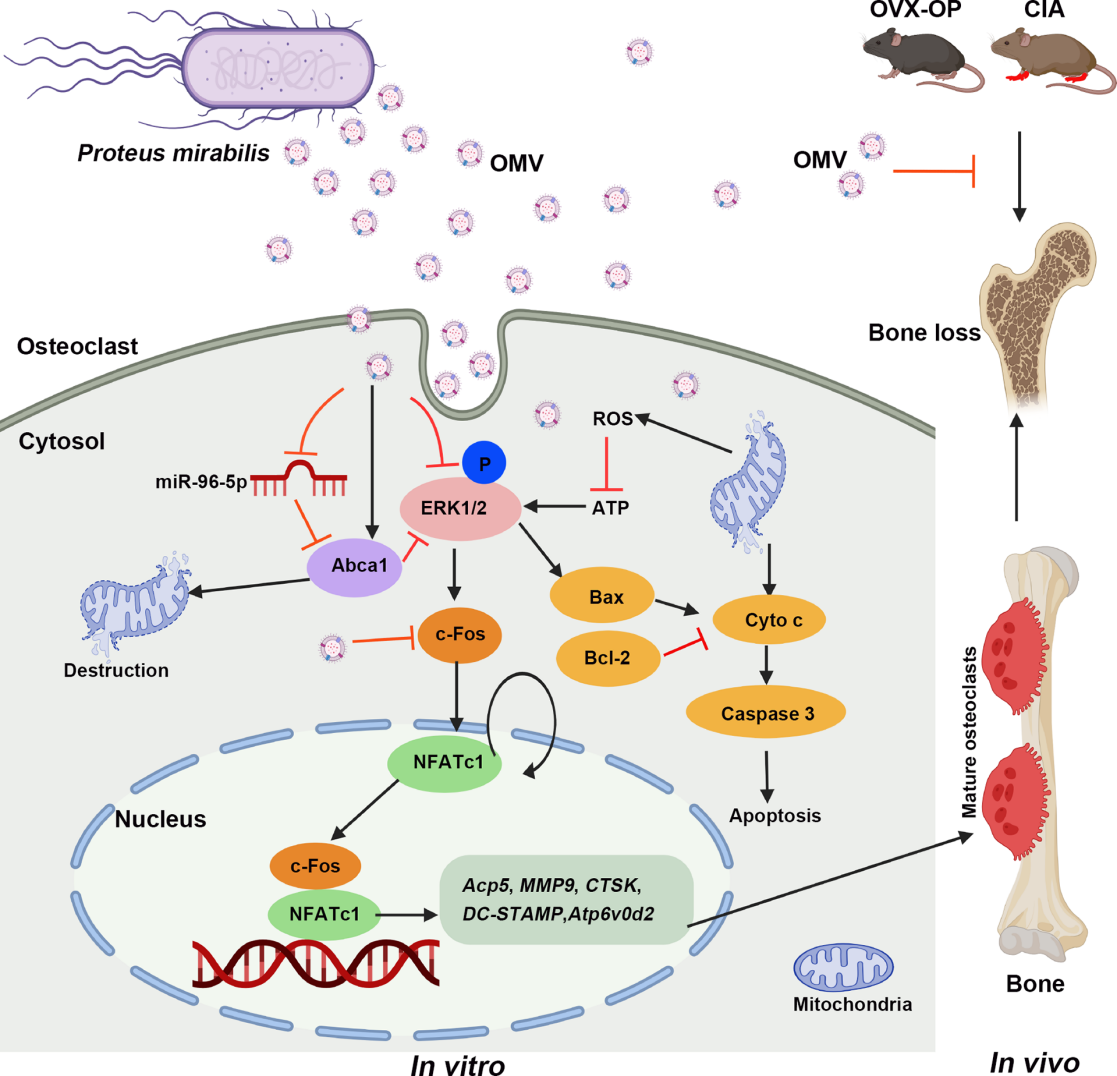
Possible molecular pathways involved in P.M OMV-mediated osteoprotection. ATP, adenosine triphosphate; Abca1, ATP binding cassette subfamily A member 1; CIA, CII-induced arthritis; Cyto c, cytochrome c; OC, osteoclast; OMV, outer membrane vesicle; OVX, ovariectomy; ROS, reactive oxygen species.

Several microRNAs affect osteoclast formation and function during bone development and degeneration by modulating their target genes (13, 15, 60, 61). Based on the interactions between miRNA and their target genes predicted using algorithms, and RT-qPCR validation results, we selected miR-96-5p for subsequent studies. Earlier miR-96-5p was shown to promote proliferation and osteogenesis of bone marrow mesenchymal stem cells (62). However, its role on osteoclasts has not been reported. Inhibition of miR-96-5p affected the expression of apoptotic proteins with an increase in ROS production, osteoclast apoptosis, and an obvious decrease in MMP, which contributed to the inhibition of osteoclast formation. In addition, downregulation of miR-96-5p has also increased the expression of its target gene Abca1 (63), which significantly improved osteoporosis (64). Our luciferase assay confirmed the negative modulation Abca1 expression by an upregulated miR-96-5p level. When osteoclasts were treated with miR-96-5p mimic and P.M OMVs together, the OMV effect on osteoclast apoptosis and Abca1 expression was counteracted suggesting miR-96-5p involvement in these processes. Earlier, Abca1 was reported to negatively regulate MAPKs and promote Cyto c release causing mitochondrial destruction and non-apoptotic/necrotic cell death (40–42). After transfection with a miR-96-5p mimic, we found a decline in p-ERK levels. Taken together, P.M OMVs inhibited osteoclast differentiation by mainly modulating the miR-96-5p/Abca1 axis, leading to mitochondria-dependent apoptosis, which provides a mechanistic basis for OMVs-mediated osteoprotective functions (**Figure 8**).

Dysregulation of OC apoptosis pathways leads to a significant increase in the number of differentiating OCs, which migrate to the surface of bones and resorb them by secreting several osteolytic enzymes (59) Moreover, OP- and RA-associated bone loss is closely related with an excessive osteoclast activity (65), and this observation is reflected well in the experimental models as well (66). Therefore, inducers of OC apoptosis are considered to be potential therapeutic agents for treating bone loss. In this study, after treatment with P.M OMVs, bone loss was significantly improved in the experimental models of OP and RA, which confirmed the osteoprotective nature of OMVs.

In summary, for the first time, we identified a new osteoprotective function for OMVs from a pathogenic bacterium and also the molecular pathways involved in it that can be exploited for clinical application to treat osteolytic patients. Use of *P. mirabilis* OMVs for therapy could be improved further by modifying the bacterial surface using genetic engineering techniques that could reduce toxicity (67, 68) while inhibiting both inflammation and bone erosion. In addition, this study could also plausibly form a basis for new investigations to identify OMVs from other unique bacterial species that will be protective against damage caused to many cells/tissues by different diseases.

## Data Availability Statement

The miRNA and mRNA sequencing data presented in the study are available in the NCBI repository, accession number: PRJNA792948. Other raw data supporting the conclusions of this article will be made available by the authors, without undue reservation, upon reasonable request.

## Ethics Statement

The animal study was reviewed and approved by the Southern Medical University Animal Ethics Board, Guangzhou, China.

## Author Contributions

TW did most of the experiments, analyzed, interpreted the data, and wrote the manuscript with input from all the other authors (QF, JO, HW, and YW). LM helped with MC3T3-E1 and NIH3T3 cultures and did part of WB experiments. KSN conceived and supervised the project, and also revised the manuscript. All the authors approved the submitted version.

## Funding

This project was supported by the grants given to KSN from Southern Medical University, China (grant numbers C1034211 and C1051004).

## Conflict of Interest

We have submitted a patent application describing the use of OMVs for the treatment of osteolytic diseases in which TW and KSN are listed as inventors.

The remaining authors declare that the research was conducted in the absence of any commercial or financial relationships that could be construed as a potential conflict of interest.

## Publisher’s Note

All claims expressed in this article are solely those of the authors and do not necessarily represent those of their affiliated organizations, or those of the publisher, the editors and the reviewers. Any product that may be evaluated in this article, or claim that may be made by its manufacturer, is not guaranteed or endorsed by the publisher.

## Glossary

**Table:**

3’-UTR	3’-untranslated regions
Abca1	ATP binding cassette subfamily A member 1
Acp5	tartrate-resistant acid phosphatase 5
ALP, AP	alkaline phosphatase
AP-1	activating protein-1
ARS	alizarin red stain
ATP	adenosine triphosphate
Bax	BCL2-associated X protein
Bcl-2	B-cell leukemia/lymphoma 2
BMMs	bone marrow-derived macrophages
BSP	bone sialoprotein
CCK-8	cell counting kit 8
CIA	collagen-induced arthritis
CTSK	cathepsin K
CTX-1	carboxy-terminal cross-linked telopeptide of type 1 collagen
cyc1	cytochrome c1
Cyto c	cytochrome c
DEGs	differentially expressed genes
*E. coli*	*Escherichia coli*
ERK	extracellular signal-regulated kinase
M-CSF	macrophage colony-stimulating factor
GO	gene ontology
H&E	hematoxylin and eosin
itgβ3	integrin subunit β 3
IκB α	I kappa B alpha
Jdp2	Jun dimerization protein 2
KEGG	Kyoto encyclopedia of genes and genomes
*L. acidophilus*	*Lactobacillus acidophilus*
*L. casei*	*Lactobacillus casei*
LPS	lipopolysaccharide
Maf	musculoaponeurotic fibrosarcoma
MAPK	mitogen-activated protein kinase
MEM	minimum essential medium
MMP (ΔΨm)	mitochondrial membrane potential
MMP9	matrix metalloproteinase 9
N.Oc/B.Pm	number of osteoclasts per bone perimeter
NFATc1	nuclear factor of activated T cells 1
NF-κB	nuclear factor kappa B
Nlgn2	neuroligin 2
OB	osteoblast
OC	osteoclast
OCN	osteocalcin
OMVs	outer membrane vesicles
OP	osteoporosis
OPN	osteopontin
OVX	ovariectomy
P.M	*Proteus mirabilis*
RA	rheumatoid arthritis
RANKL	receptor activator of nuclear factor kappa-B ligand
RLU	relative light unit
ROS	reactive oxygen species
Runx2	runt-related transcription factor 2
SIRT1	sirtuin 1
TEM	transmission electron microscope
TRAP	tartrate-resistant acid phosphatase
Uqcrc2	ubiquinol-cytochrome c reductase core protein 2
Zhx2	zinc fingers and homeoboxes 2
