# Supplementary material for: Proteus mirabilis Vesicles Induce Mitochondrial Apoptosis by Regulating miR96-5p/Abca1 to Inhibit Osteoclastogenesis and Bone Loss
Source: Front Immunol. 2022 Feb 15;13:833040. doi: 10.3389/fimmu.2022.833040 (PMC8885728; doi:10.3389/fimmu.2022.833040)
Supplement: Supplementary file 1 [file DataSheet_1.pdf]

Supplementary Information

Supplementary Table 1. The mRNA sequence list

Genes name	Primer sequence (5'-3') Forward	Primer sequence (5'-3') Reverse
Acp5	TCATGGGTGGTGCTGCT	GCCCACAGCCACAAATCT
MMP9	AAGGCAGCGTTAGCCAGAA	GCGGTACAAGTATGCCTCTGC
CTSK	GTGGATGAAATCTCTCGGCGT	CACTGGTCAT GTCTCCCAAGT
NFATc1	TGTTGCTCTGGGGTGTATC	CATGTGCCCTGGAGAGTT
c-Fos	CAGCGAGCAACTGAGAAGA	AAGGGGTCCAGGGGTAG
Itgβ3	CAGTGGCCGGGACAACTC	GACAAAGTCTCATCTGAGCACCAG
Jdp2	AGAAGGAACGCACAGAGTTTC	TGAGCATCAGGATAAGCTGTTG
Runx2	GCCGGGAATGATGAGAAC	TGGGGAGGATTTGTGAAGA
OCN	CAGTCCCCAGCCCAGAT	GCGTTTGTAGGCGGTCTT
OPN	GAGCGAGGATTCTGTGGA	TCGACTGTAGGGACGATTG
BSP	AAGCAGGTGCAGAAGGAA	GCGAGGTGGTCCCATAG
Cyc1	ACATCGTTCGAGCTAGGCAT	CTGGGGTGCCATCATCATACT
Uqcrc2	GGATCTTGAGTTTACCAAATTACC	AGATGAAGCTCCTTTGGTAGTC
Abca1	CTTCCCACATTTTTGCCTGG	AAGGTTCCGTCCTACCAAGTCC
β-actin	CCTCTATGCCAACACAGTGC	CCTGCTTGCTGATCCACATC

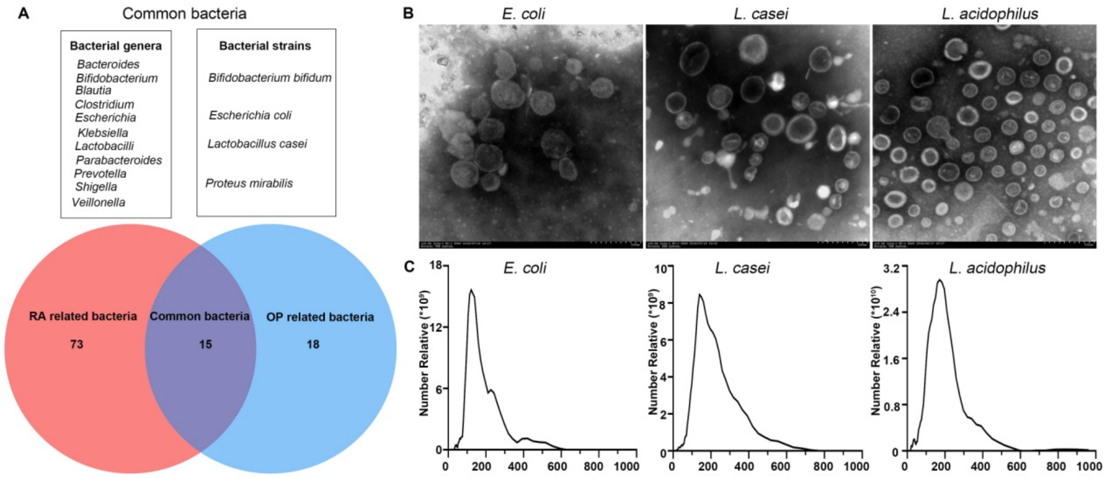

Supplementary Figure 1. Characterization of OMVs from commonly identified bacteria from RA and OP. (A) Bacteria shared between rheumatoid arthritis and osteoporosis from MDIDB website. **(B)** Morphology and **(C)** size determination of *E. coli*, *L. casei* and *L. acidophilus* by transmission electron microscopy and nanotrack analysis. *E. coli*: *Escherichia coli*; *L. casei*: *Lactobacillus casei*; *L. acidophilus*: *Lactobacillus acidophilus*. Scale bar = 200 nm. Data are from three independent experiments.

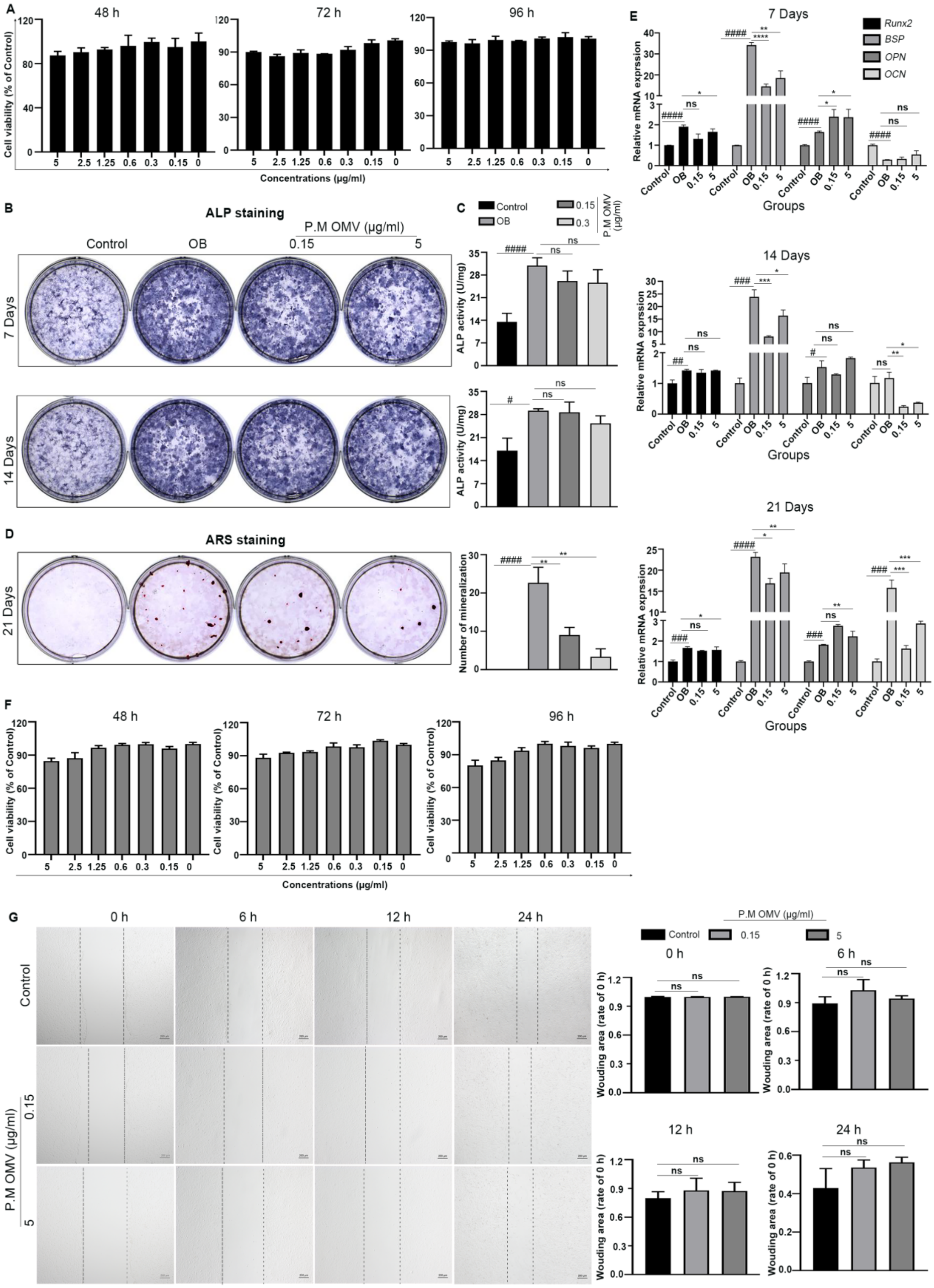

Supplementary Figure 2. Differential effects of P.M OMVs on osteoblasts and fibroblasts. (A) P.M OMVs had minimal effect on osteoblast (MC3T3-E1) cell viability, **(B-C)** ALP activity on day 7 and 14. However, **(D)** mineralized nodes (alizarin red stain) were significantly affected after 21 days. **(E)** P.M OMVs differentially affected the expression of OB-related genes (*Runx2*, *BSP*, *OPN* and

OCN) on various days. Negligible effect of P.M OMVs on **(F)** cell viability and **(G)** migration ability of fibroblasts. P.M: *Proteus mirabilis*. scale bar = 200 μm. Data are from three independent experiments and represented as mean ± SD. #, $p < 0.05$; ##, $p < 0.01$; ###, $p < 0.001$; ####, $p < 0.0001$ compared to Control. *, $p < 0.05$; **, $p < 0.01$; ***, $p < 0.001$; ****, $p < 0.0001$ to OB. ns, not significant.

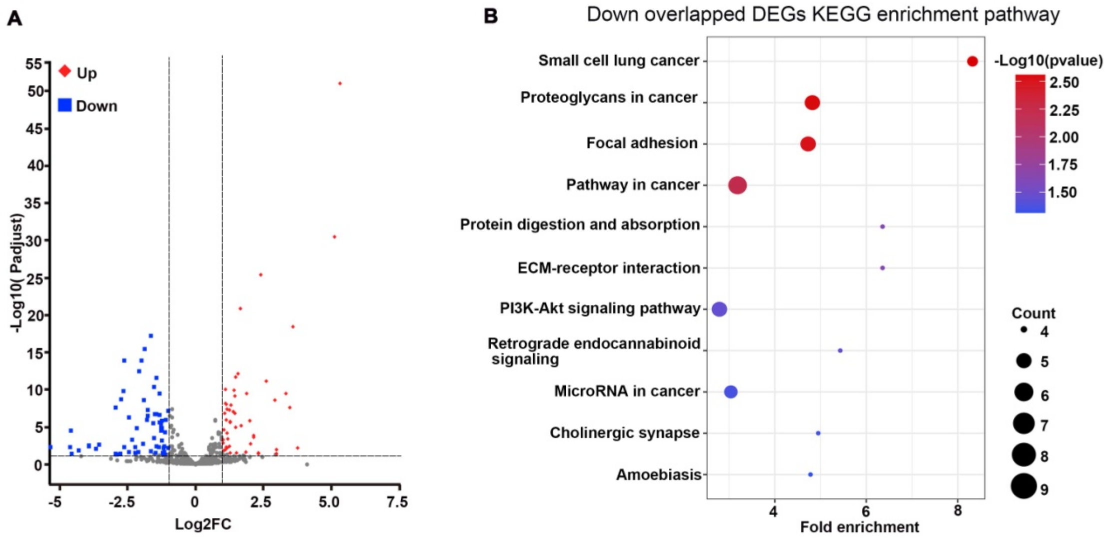

Supplementary Figure 3. Volcano plot of miRNA and the KEGG enrichment analysis of overlapped DEGs. (A) Volcano plot of miRNA genes altered by RANKL (log2 FC ⩾ 1, $p < 0.01$), **(B)** KEGG enrichment analysis of down-regulated genes are shown in the bubble chart. The top 15 genes that were significantly enriched in the KEGG pathway ($p < 0.05$) are presented.

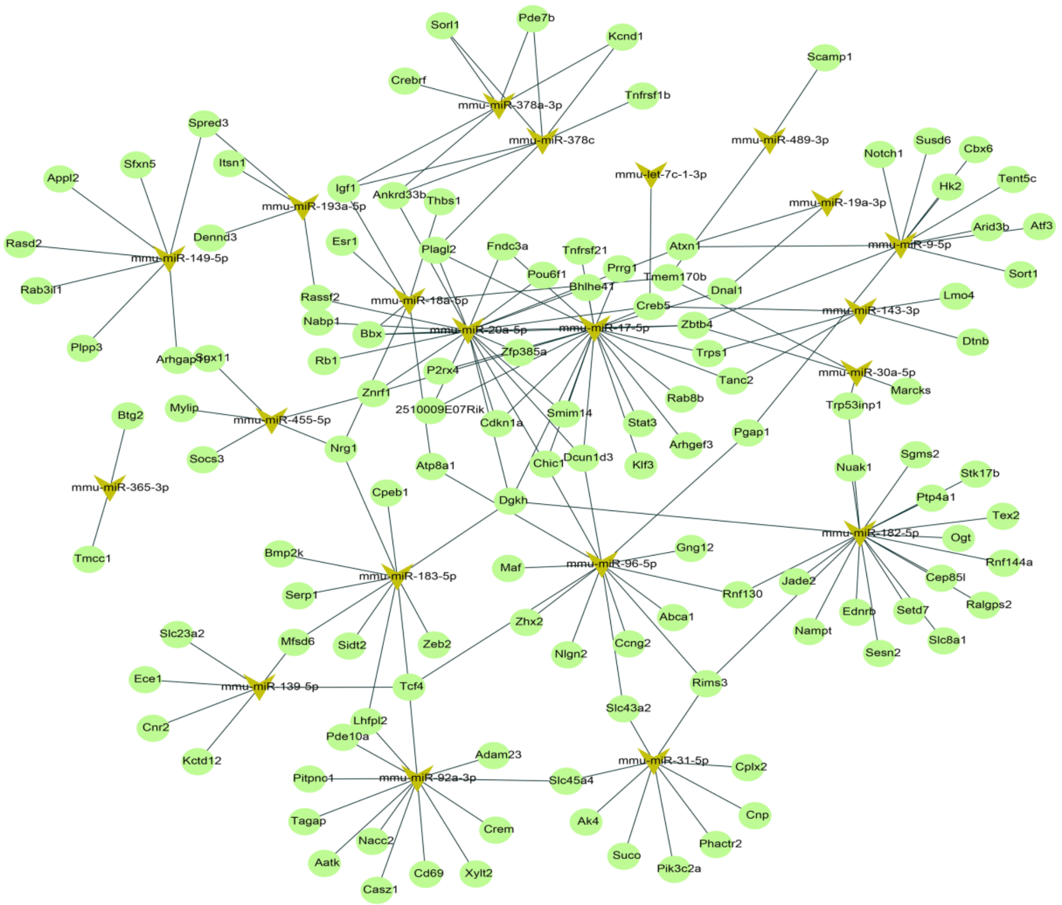

Supplementary Figure 4. The network of down-regulated miRNA-mRNA. The regulatory network of 177 down- regulated miRNA-mRNA pairs.

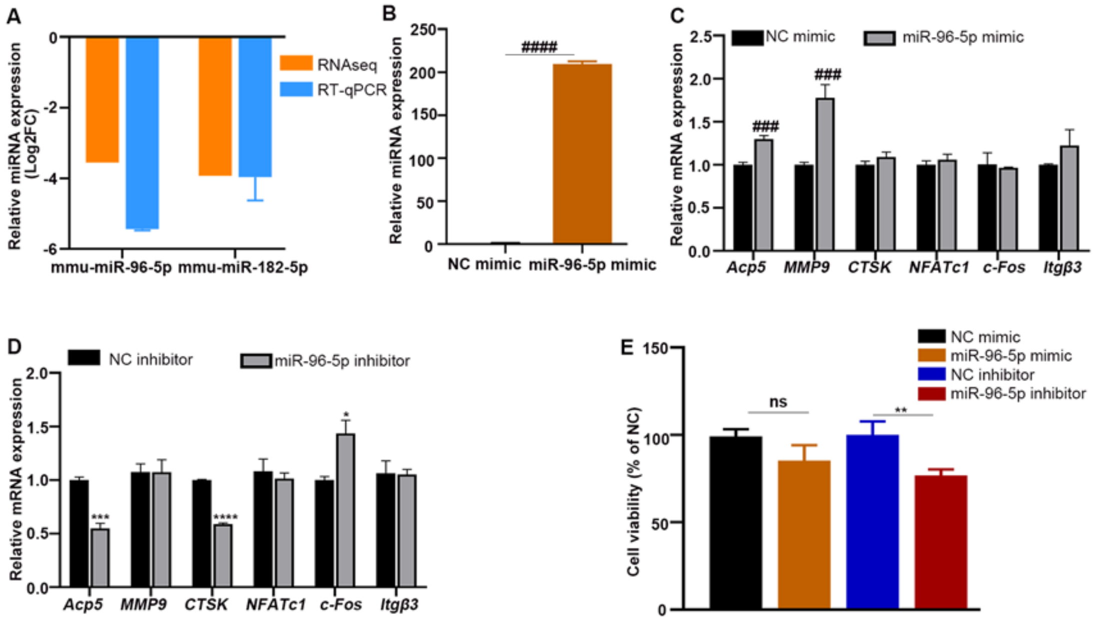

Supplementary Figure 5. The effect of miR-96-5p on osteoclasts. (A) Relative miRNA expression from P.M OMVs or RANKL treated groups. **(B)** Transfection efficiency of miR-96-5p. **(C-D)** Effect of mmu-miR-96-5p mimic or inhibitor on OC-related genes and **(E)** osteoclast viability. ###, $p < 0.001$; ####, $p < 0.0001$ compared to NC mimic. *, $p < 0.05$; **, $p < 0.01$; ***, $p < 0.001$; ****, $p < 0.0001$ compared to NC inhibitor. ns, not significant.

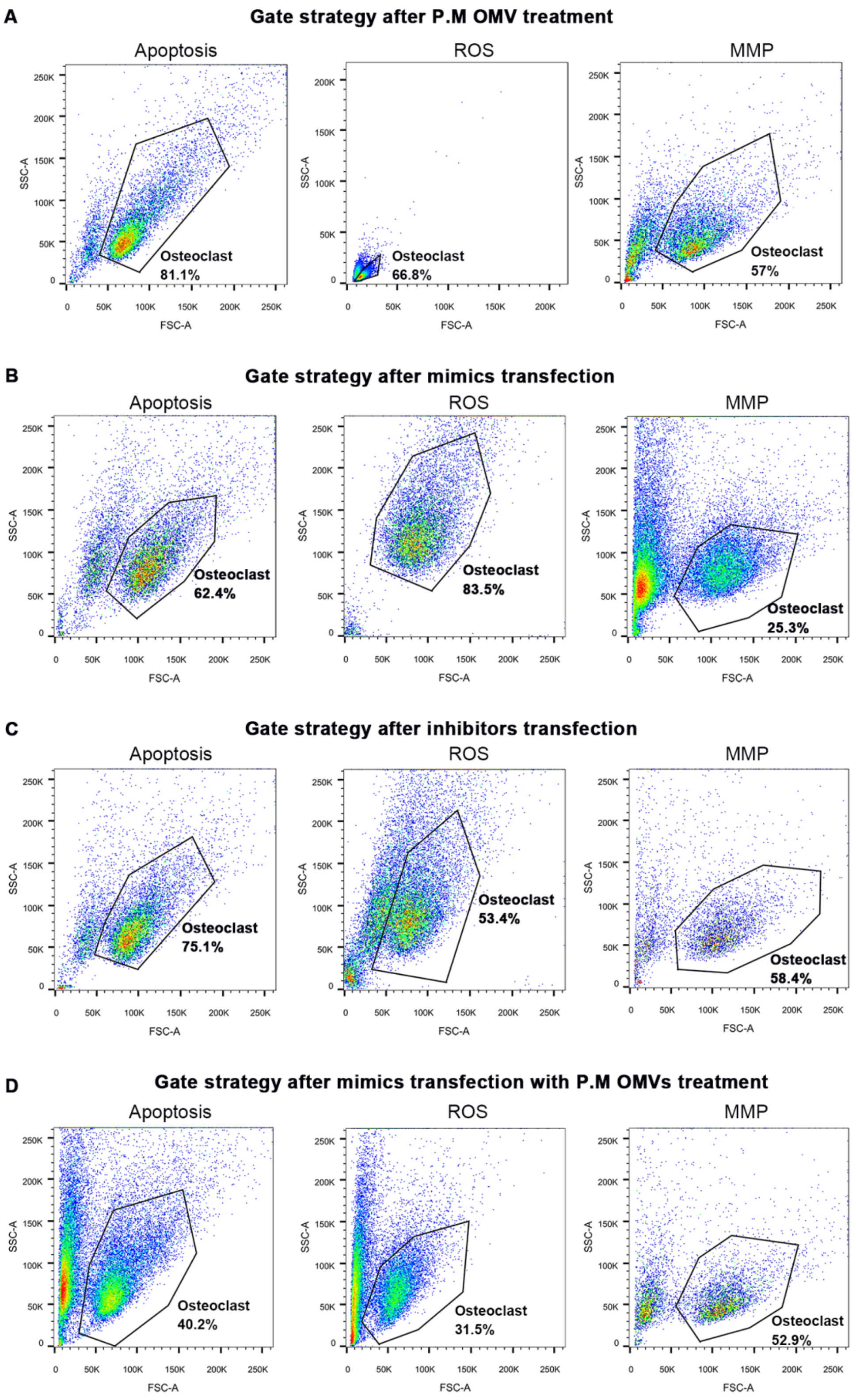

Supplementary Figure 6. FACS gating strategy. (A) gating strategy for apoptosis, for ROS and for MMP after P.M OMV treatment and **(B)** mimics and **(C)** inhibitor transfection and **(D)** mimics transfection with P.M OMVs treatment.

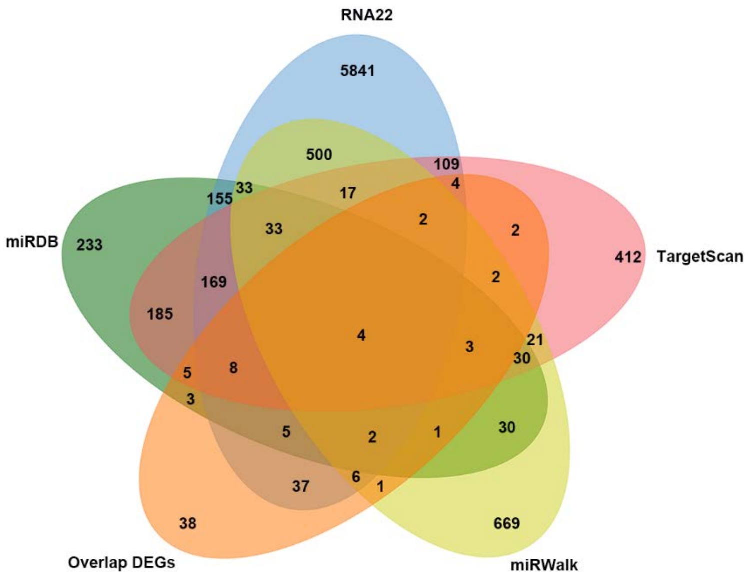

Supplementary Figure 7. Prediction of miR-96-5p target genes. The intersection of mmu-miR-96-5p target genes predicted by TargetScan, RNA22, miRDB and miRWalk with the overlaped target DEGs of miR-96-5p from RNAseq data.

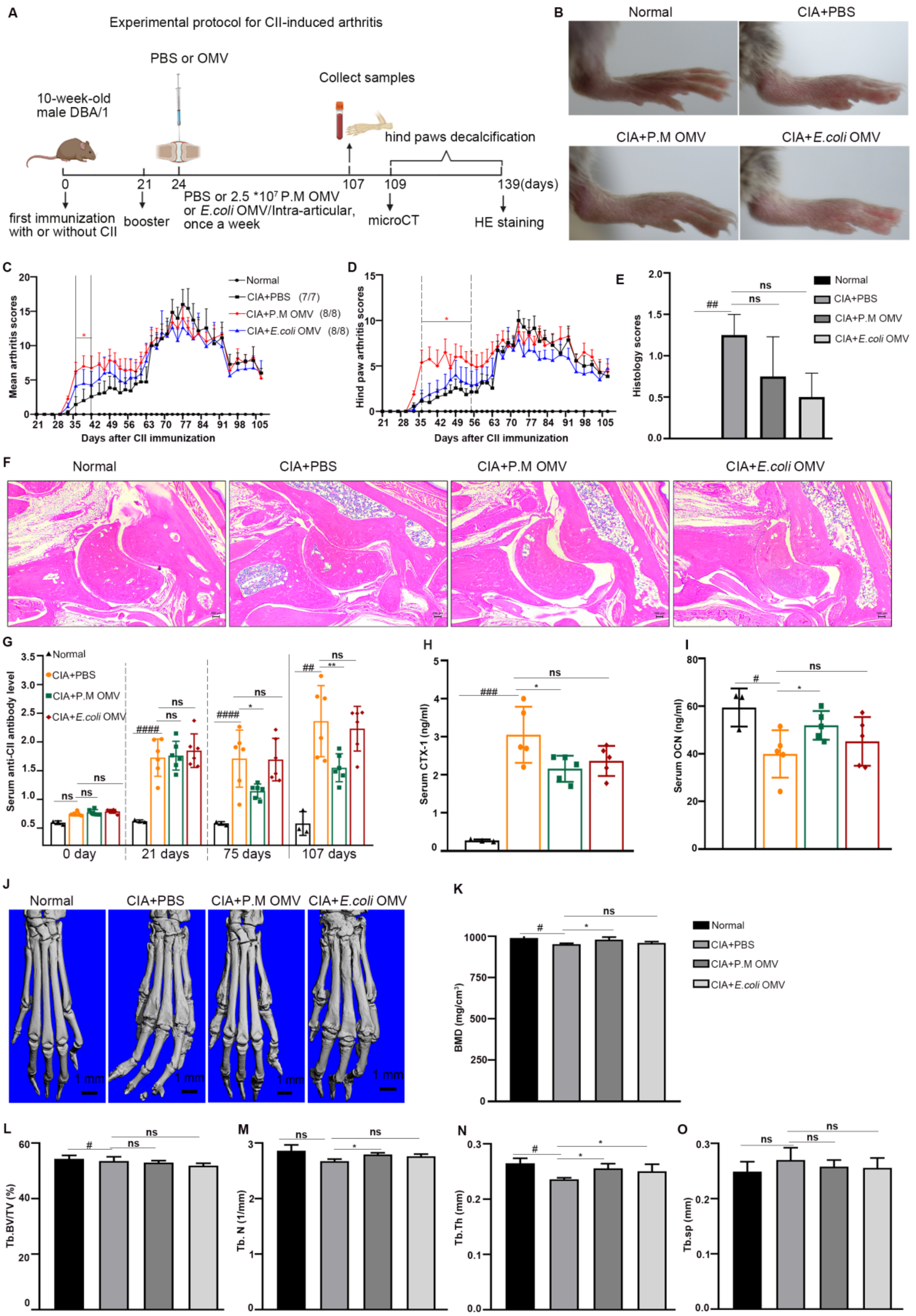

Supplementary Figure 8. Effect of P.M OMVs on inflammation and bone erosion in CIA. (A) Experimental protocol. **(B)** Representative pictures of hind paws of mice from different groups. **(C-D)** Mean arthritis and hind paw arthritis scores from each group after booster immunization. **(E-F)** HE staining of paw sections on day 107. **(G)** Serum anti-CII antibody levels in each group on days 0, 21, 75 and 107 (normal group

n = 3, other groups n = 5). Serum **(H)** CTX-1 and **(I)** OCN levels. **(J)** Representative images of hind paws after μCT (n = 3). **(K-O)** Analysis of bone parameters in different groups. n, indicates number of mice. BMD: bone marrow density, Tb.BV/TV: trabecular bone volume per tissue volume, Tb.N: trabecular number, Tb.Sp: trabecular separation and Tb.Th: trabecular thickness.. Arthritis scores are represented as mean ± SEM, and the remaining data are given as mean ± SD. #, $p < 0.05$; ##, $p < 0.01$, ###, $p < 0.001$, ####, $p < 0.0001$ compared to Normal; *, $p < 0.05$; **, $p < 0.01$compared to CIA. ns, not significant.
